# Comparative *in vivo *gene expression of the closely related bacteria *Photorhabdus temperata *and *Xenorhabdus koppenhoeferi *upon infection of the same insect host, *Rhizotrogus majalis*

**DOI:** 10.1186/1471-2164-10-433

**Published:** 2009-09-15

**Authors:** Ruisheng An, Srinand Sreevatsan, Parwinder S Grewal

**Affiliations:** 1 Department of Entomology, The Ohio State University, Wooster, OH 44691, USA; 2 Department of Veterinary Population Medicine, University of Minnesota, St. Paul, MN 55108, USA

## Abstract

**Background:**

*Photorhabdus *and *Xenorhabdus *are Gram-negative, phylogenetically related, enterobacteria, forming mutualism with the entomopathogenic nematodes *Heterorhabditis *and *Steinernema*, respectively. The mutualistic bacteria living in the intestines of the nematode infective juveniles are pathogenic to the insect upon release by the nematodes into the insect hemolymph. Such a switch needs activation of genes that promote bacterial virulence. We studied *in vivo *gene expression in *Photorhabdus temperata *and *Xenorhabdus koppenhoeferi *upon infection of the white grub *Rhizotrogus majalis *using selective capture of transcribed sequences technique.

**Results:**

A total of 40 genes in *P. temperata *and 39 in *X. koppenhoeferi *were found to be upregulated in *R. majalis *hemolymph at 24 h post infection. Genomic presence or upregulation of these genes specific in either one of the bacterium was confirmed by the assay of comparative hybridization, and the changes of randomly selected genes were further validated by quantitative real-time PCR. The identified genes could be broadly divided into seven functional groups including cell surface structure, regulation, virulence and secretion, stress response, intracellular metabolism, nutrient scavenging, and unknown. The two bacteria shared more genes in stress response category than any other functional group. More than 60% of the identified genes were uniquely induced in either bacterium suggesting vastly different molecular mechanisms of pathogenicity to the same insect host. In *P. temperata lysR *gene encoding transcriptional activator was induced, while genes *yijC *and *rseA *encoding transcriptional repressors were induced in *X. koppenhoeferi*. Lipopolysaccharide synthesis gene *lpsE *was induced in *X. koppenhoeferi *but not in *P. temperata*. Except *tcaC *and *hemolysin *related genes, other virulence genes were different between the two bacteria. Genes involved in TCA cycle were induced in *P. temperata *whereas those involved in glyoxylate pathway were induced in *X. koppenhoeferi*, suggesting differences in metabolism between the two bacteria in the same insect host. Upregulation of genes encoding different types of nutrient uptake systems further emphasized the differences in nutritional requirements of the two bacteria in the same insect host. *Photorhabdus temperata *displayed upregulation of genes encoding siderophore-dependent iron uptake system, but *X. koppenhoeferi *upregulated genes encoding siderophore-independent ion uptake system. *Photorhabdus temperata *induced genes for amino acid acquisition but *X. koppenhoeferi *upregulated *malF *gene, encoding a maltose uptake system. Further analyses identified possible mechanistic associations between the identified gene products in metabolic pathways, providing an interactive model of pathogenesis for each bacterium species.

**Conclusion:**

This study identifies set of genes induced in *P. temperata *and *X. koppenhoeferi *upon infection of *R. majalis*, and highlights differences in molecular features used by these two closely related bacteria to promote their pathogenicity in the same insect host.

## Background

Entomopathogenic Gram-negative enterobacteria *Photorhabdus *and *Xenorhabdus *form symbioses with the entomopathogenic nematodes *Heterorhabditis *and *Steinernema*, respectively [1]. These bacteria not only have similar biology but are also phylogenetically related based on 16s rDNA sequence identities [2]. They naturally colonize intestines of the nematode infective juveniles which invade susceptible insects to release the bacteria into the hemolymph. The bacteria multiply in the hemolymph, killing the insect host within 24-48 h and converting the cadaver into a food source suitable for nematode growth and reproduction. After 1-3 rounds of nematode reproduction, the bacteria recolonize the emerging infective juveniles ensuring their transmission to a new host [1].

Available evidence suggests that *Photorhabdus *and *Xenorhabdus *encode specific factors to engage in a pathogenic relationship with the insect host [3]. The published genome sequence of *Photorhabdus luminescens *TT01 strain indicates that virulence genes are encoded within a number of pathogenicity islands located on the bacterial chromosome [4,5]. Besides producing toxins to cause insect death, *Photorhabdus *and *Xenorhabdus *have to first evade the insect's immune response to establish a successful infection. The two bacteria differ in mechanisms by which they evade host immune responses. For example, in *Photorhabdus*, mutational inactivation of *phoP *gene results in increased sensitivity to insect immune response and decreased virulence towards insects [6,7], while in *X. nematophila*, *phoPQ *mutants are more susceptible to immune response but are fully virulent [3]. *P. luminescens *produces a signaling molecule AI-2 to resist reactive oxygen species [8] and phenylpropanoid chemical ST to inhibit the activity of antimicrobial enzyme PO and formation of melanotic nodules [9], but the strategy used by *X. nematophila *appears to be that of suppression of transcripts involved in the insect immune response [10-12]. In addition, *P. luminescens *encodes a type III secretion system and one of the effectors, LopT, suppresses phagocytosis and reduces nodulation by haemocytes [13,14]. However, the genomes of *Xenorhabdus bovienii *and *X. nematophila *do not show homologues of LopT or a dedicated type III secretion system [3], and *Xenorhabdus *likely uses flagellar regulatory cascade to control cellular immunity [15].

Despite the identification of the above regulatory and virulence factors from *Photorhabdus *and *Xenorhabdus*, the full profiles of genes expressed during insect infection by these two closely related bacteria are unknown. Münch et al. [16] identified 29 promoters induced by *P. luminescens *in the presence of isolated *Galleria mellonella *homogenate using a differential fluorescence induction approach. However, treatment with insect homogenate might neglect physicochemical parameters as inducers which could result in different sets of genes being upregulated during natural infection of live insects. We used selective capture of transcribed sequences (SCOTS) technique to study *in vivo *gene expression in *Photorhabdus temperata *(associated with the nematode *Heterorhabditis bacteriophora *GPS11) and *Xenorhabdus koppenhoeferi *(associated with the nematode *Steinernema scarabaei *AMK001) during infection of the same insect host *Rhizotrogus majalis*. They were selected according to their distinct pathogenicity in insects of the family of Scarabaeidae - *S. scarabaei *is more virulent to *R. majalis *than *H. bacteriophora*. However, unlike their nematode partners, both bacterial species are highly virulent to *Rhizotrogus majalis*. In addition, although *X. koppenhoeferi *grows slower than *P. temperata *both *in vitro *and *in vivo*, the LD50 and LT50 value do not differ between these two bacteria [17]. We hypothesize that the two bacteria species will use both common and distinct molecular mechanisms during infection of the same insect host.

## Results

### Isolation of bacterial transcripts induced in Rhizotrogus majalis

**Figure 1:**
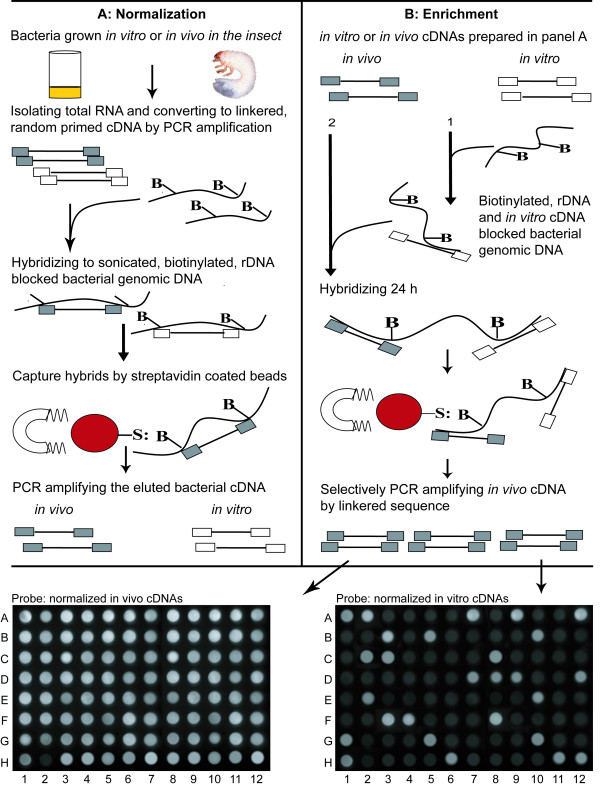
**Schematic presentation of the Selective Capture of Transcribed Sequences (SCOTS) technique followed by Southern blot analysis of SCOTS identified sequences**. In panel A, normalized bacterial cDNAs were obtained directly from bacteria grown *in vitro *in the Brain Heart Infusion broth or *in vivo *in the infected insect *Rhizotrogus majalis*. In panel B, cDNAs corresponding to genes preferentially expressed in *R. majalis *relative to the broth were enriched by differential cDNA hybridization. The enriched cDNAs were transformed into a cloning vector to build the cDNA library. Cloned inserts were amplified by PCR, equally transferred to two nylon membranes, and probed with digoxigenin labeled normalized *in vivo *cDNAs (left) or normalized *in vitro *cDNAs (right) as described in Methods. The dots at the same position in the two arrays were loaded with the same amplicon of each individual clone from the enriched cDNA library, and the concentration of probes was standardized to be same.

To isolate bacterial genes induced upon infection, total RNA was isolated from live grubs at 24 h post injection with *P. temperata *or *X. koppenhoeferi*, as well as from 48 h log-phase bacterial cultures grown in Brain Heart Infusion (BHI) broth that were used to inject *R. majalis*. Random-primed bacterial cDNAs were normalized by hybridization to biotinylated bacterial genomic DNA that had been blocked beforehand using bacterial ribosomal RNA operon, resulting in sampling of bacterial mRNA transcripts apart from its ribosomal and insect transcripts. The normalized bacterial cDNAs representing total mRNA transcripts produced by bacteria grown *in vitro *or within the infected insects were named as normalized *in vitro *and *in vivo *cDNA libraries, respectively. To isolate bacterial mRNA transcripts preferentially induced during infection of the insect compared to the culture, normalized *in vivo *cDNAs were enriched by hybridization to biotinylated bacterial genomic DNA that had been pre-hybridized with rRNA operon and *in vitro *normalized cDNAs. The enriched cDNAs representing *in vivo*-induced genes, which were either lower in abundance or absent in 48 h *in vitro *bacterial cultures were then cloned into a TA cloning vector to construct *in vivo *enriched cDNA libraries. A total of 384 clones (192 for each bacterium) derived from enriched cDNA libraries were randomly picked for screening. Out of these, the clones (150 for *P. temperata *and 140 for *X. koppenhoeferi*) that showed stronger signal upon hybridization with *in vivo*-derived cDNAs compared to *in vitro*-derived cDNAs (Fig. 1) were sequenced and analyzed using the non-redundant algorithms of BLAST in NCBI and database of *Xenorhabdus *Genomes. These sequenced clones were therefore considered to represent reliable transcripts specifically expressed in *P. temperata *or *X. koppenhoeferi *after 24 h infection of *R. majalis*.

### Analysis of bacterial genes induced in Rhizotrogus majalis

**Figure 2:**
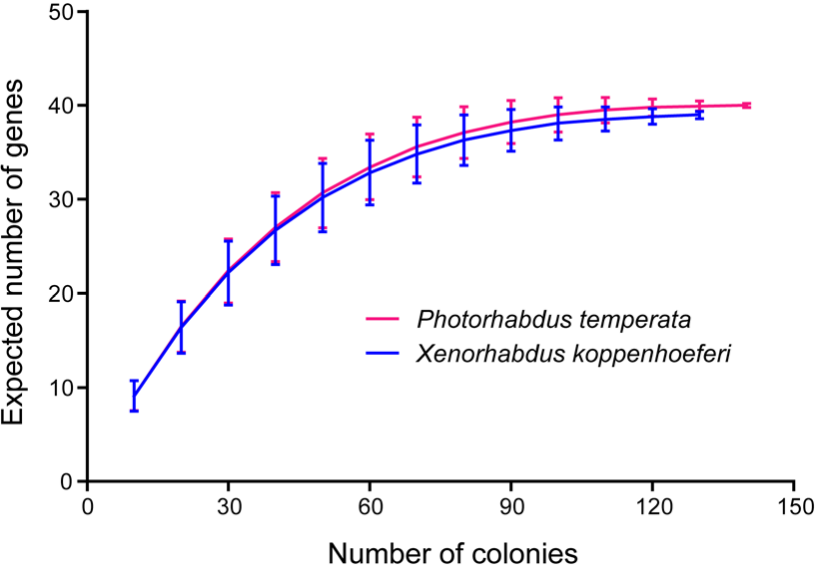
**Rarefaction analysis curves demonstrating coverage of cDNA libraries for genes upregulated in bacteria *Photorhabdus temperata *or *Xenorhabdus koppenhoeferi *during infection of the insect *Rhizotrogus majalis***.

**Figure 3:**
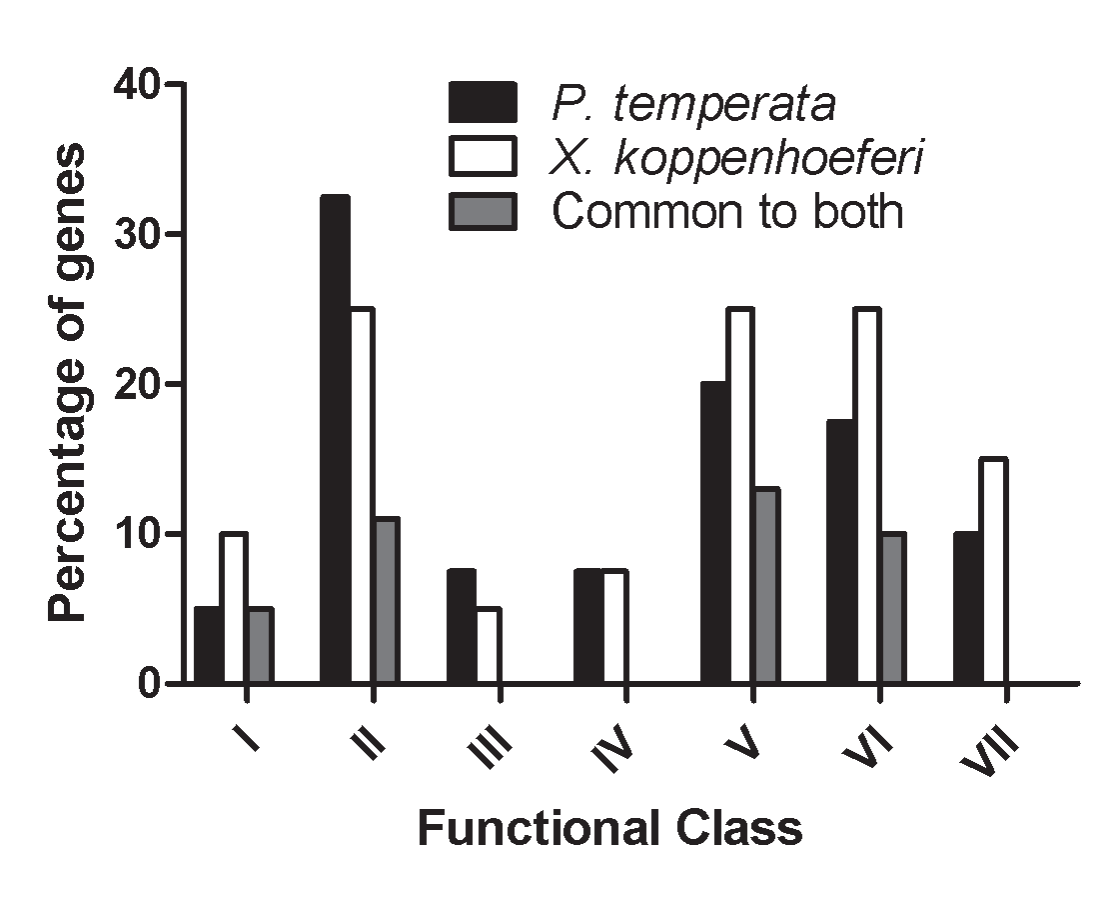
**Distribution of SCOTS isolated *in vivo*-induced *Photorhabdus temperata *and *Xenorhabdus koppenhoeferi *genes among different functional classes: cell structure (I), intracellular metabolism (II), nutrient scavenging (III), regulation (IV), adaptation to environmental stresses (V), virulence and secretion (VI), and unknown function**. The percentage of genes for *P. temperata *or *X. koppenhoeferi *was presented as ratio of the number of genes in each functional class to the total number of genes from the respective bacterium, and the percentage of genes for common to both was calculated as ratio of the number of common genes in each functional class to the sum of genes from both bacteria.

Most identified transcripts were either similar to sequences from *P. luminescens *or *X. nematophila*, suggesting that bacterial transcripts were successfully enriched from the infected insects. For both bacteria, each isolated transcript was identified at least twice from the sequenced clones. The coverage of *in vivo *enriched libraries was then evaluated using Analytic Rarefaction according to the redundancy of each identical transcript in the sequenced clones. As indicated by the rarefaction curves (Fig. 2), saturation was achieved for the sequenced clones, indicating that most of representative genes in the *in vivo *enriched cDNA libraries were identified. Totally, we isolated 40 different sequences from *P. temperata *(Table 1) and 39 from *X. koppenhoeferi *(Table 2). Based on homology searches, the genes corresponding to distinct sequences could be divided into seven functional groups: cell surface structure (Class I), intracellular metabolism (Class II), nutrient scavenging (Class III), regulation (Class IV), stress response (Class V), virulence (Class VI) and uncharacterized genes (Class VII). Genes involved in metabolism constitute about 25% to 33% of the total number of *in vivo*-induced genes (Fig. 3), showing large numbers of metabolic changes during insect infection. Semi-quantitative reverse transcriptase PCR performed on 10 randomly selected identified genes (five from *P. temperata*: *cysK*, *fliA*, *lysR*, *pchC *and *pmt1 *and five from *X. koppenhoeferi*: *aceE*, *dacC*, *cobJ*, *malF*, and *yijC*) showed that these genes were detectable from the *in vivo*-derived cDNA but not from *in vitro*-derived cDNA. In addition, 18S rRNA gene, a eukaryotic housekeeping gene, was detected in the *in vivo *cDNA population before normalization, but not after normalization, suggesting that bacterial cDNAs were successfully purified from the insect cDNAs after SCOTS normalization. Also gyrase A gene, a prokaryotic housekeeping gene, was not detected from the enriched *in vivo *cDNAs, but was detected in all other cDNA populations including *in vitro *and *in vivo *cDNAs before and after normalization, indicating that only differentially expressed genes were captured after enrichment.

**Table 1:** SCOTS identified *Photorhabdus temperata *genes induced upon infection of the white grub *Rhizotrogus majalis*.

**Class**	**Gene --Possible function**	**Id/Sim%**	**Span**	**E-value**
**Cell struture**				
	***skp***-Putative outer membrane protein (*Photorhabdus luminescens*, plu0681)	91/98	165	1E-11
	***fliA***-Flagella biosynthesis sigma factor (*P. luminescens*, plu1955)	98/100	150	8E-20
**Regulation**				
	***cpxR***-Response regulator (*P. luminescens*, plu4794)	100/100	105	1E-14
	***lysR***-Transcriptional activator protein (*P. luminescens*, plu1190)	90/96	210	7E-30
	***phoP***-Response regulator (*P. luminescens*, plu2807)	95/98	444	1E-89
**Virulence**				
	***fhaC***-Putative hemolysin secretion protein (*P. luminescens*, plu3065)	96/96	90	1E-09
	***sctL***-Putative type III secretion system (*P. luminescens*, plu3787)	89/92	341	3E-50
	***pmt1***-Similar to macrophage toxin Pmt1 (*P. luminescens*, plu0359)	92/92	78	2E-5
	***pplA***-Putative phospholipase A (*P. luminescens*, plu3370)	100/100	105	6E-16
	***tcaC***-Insecticidal toxin complex (*P. luminescens*, plu0515)	91/94	102	5E-21
	***toxC***-Putative insecticidal toxin (*P. luminescens*, plu4092)	96/96	82	8E-09
	***virB***-Putative plasmid-related protein (*Serratia proteamaculans*, Spro_4169)	74/86	201	3E-24
**Stress response**				
	***gshB***-Glutathione synthetase (*Yersinia pseudotuberculosis*, YPTS_3340)	100/100	94	1E-11
	***dnaK***-Heat shock protein (*Vibrio cholerae*, VC0395_A0382)	98/100	141	1E-17
	***surA***-Peptidyl-prolyl cis-trans isomerase (*P. luminescens*, plu0611)	90/93	195	3e-26
	***uspB***-Universal stress protein (*Escherichia coli*, ECH74115_4840)	83/95	119	1E-10
	***dnaB***-Replicative DNA helicase (*P. luminescens*, plu4359)	99/100	306	4E-48
	***ptIS***-Putative IS4-family transposase (*Shewanella denitrificans*, Sden_1127)	61/76	210	1E-20
	***rpoB***-DNA-directed RNA polymerase beta subunit (*P. luminescens*, plu0439)	93/98	159	3E-21
	***topA***-DNA topoisomerase I (*P. luminescens*, plu2435)	88/97	104	7E-14
**Metabolism**				
- Amino acid synthesis	***cysK***-Cysteine synthase A/O-acetylserine sulfhydrolase A (*P. luminescens*, plu1395)	96/99	201	1E-20
- Amino acid metabolism	***selD***-Selenophosphate synthetase (*P. luminescens*, plu2551)	90/95	291	1E-43
- Amino acid tRNA synthesis	***def***-Peptide deformylase/N-formylmethionylaminoacyl-tRNA deformylase (*P. luminescens*, plu4695)	94/97	201	2E-27
- Cofactor biosynthesis	***bioB***-Biotin synthetase (*P. luminescens*, plu1485)	100/100	90	3E-9
- Energy metabolism	***cyoB***-Copper-type cytochrome O ubiquinol oxidase subunit I (*Yersinia enterocolitica*, YE3142)	95/100	180	5E-28
	***yncB***-NADPH: quinone reductase and related Zn-dependent oxidoreductases (*E. coli*, EFER_3231)	77/91	159	8E-18
- Fatty acid synthesis	***fabA***-Beta-ketoacyl synthase (*Saccharopolyspora erythraea*; SACE_0023)	44/63	237	9E-18
- Glucose metabolism	***kdgK***-2-dehydro-3-deoxygluconokinase (*P. luminescens*, plu0177)	81/92	111	7E-13
- Nucleotide synthesis	***purH***-Bifunctional phosphoribosylaminoimidazolecarboxamide formyltransferase/IMP cyclohydrolase (*P. luminescens*, plu0495)	86/92	213	6E-28
- Protein synthesis	***glnD***-PII uridylyl-transferase (*P. luminescens*, plu0670)	98/100	144	1E-21
	***lspA***-Signal peptidase II (*P. luminescens*, plu0592)	35/36	110	3E-14
- Protein folding	***trxA***-Thioredoxin (*Vibrio fischeri*, VF_2461)	83/98	159	4E-18
- TCA cycle	***pckA***-Phosphoenolpyruvate carboxykinase (*P. luminescens*, plu0100)	93/97	300	9E-48
**Nutrition**				
- Amino acid acquisition	***artM***-Putative ABC amino acid transport system (*Yersinia pestis*; YPDSF_3926)	86/95	168	2E-20
- Ion uptake	***pchC***-Putative pyochelin siderophore biosynthetase (*Y. pseudotuberculosis*, YpsIP31758_0684)	70/78	216	3E-26
- Importing system	***tolB***-Periplasmic component of the Tol biopolymer transport system (*P. luminescens*, plu1455)	96/99	213	8E-33
**Unknown**				
	***ptst1***-Hypothetical protein (*P. luminescens*, plu3488)	91/97	190	5E-11
	***ptst2***-Hypothetical protein (*P. luminescens*, plu2670)	75/85	177	1E-16
	***ptst3***-No similarity to known genes			
	***ptst4***-No similarity to known genes			

Underlined genes were also upregulated in *Xenorhabdus koppenhoeferi *during infection of *R. majalis*. Other genes were exclusively induced in *P. temperata *and similar sequences of double-underlined genes were absent in the genome of *X. koppenhoeferi*.

**Table 2:** SCOTS identified *Xenorhabdus koppenhoeferi *genes induced upon infection of the white grub *Rhizotrogus majalis*.

**Class**	**Gene --Possible function**	**Id/Sim%**	**Span**	**E-value**
**Cell struture**				
	***dacC***-Penicillin-binding protein 6 precursor/D-alanyl-D-alanine carboxypeptidase fraction C (*Photorhabdus luminescens*, plu1573)	65/78	330	9E-35
	***fliM***-Flagella MS-ring protein (*P. luminescens*, plu1941)	90/98	244	7E-32
	*lpsE *-Putative LPS biosynthesis protein (*P. luminescens*, plu4861)	84/86	132	9E-18
	***ompF***-Outer membrane protein (*Xenorhabdus nematophila*, XENPROT)	88/91	227	2E-38
**Regulation**				
	***rseA***-Sigma E factor negative regulator (*Serratia proteamaculans*, Spro_3674)	66/80	165	7E-16
	***tilS***-Putative cell cycle protein (*P. luminescens*, plu0689)	62/81	189	4E-14
	***yijC***-HTH-type transcriptional repressor (*Escherichia coli*, EcSMS35_4410)	100/100	129	3E-16
**Virulence**				
	***virH***-Putative toxin secretion transporter (*Vibrio cholerae*, VC0395_A1056)	94/98	230	3E-39
	***xhlA***-Putative hemolysin protein (*X. nematophila*, AY640584)	75/86	258	4E-43
	***rtxC***-RTX toxin activating protein (*Vibrio vulnificus*, VVA1030)	77/93	210	5E-23
	***tcaC***-Putative insecticidal toxin complex protein (*X. nematophila*, AJ308438)	90/95	132	9E-21
**Stress response**				
	***gor***-Glutathione reductase (*Yersinia pseudotuberculosis*, YPTS_4032)	88/94	182	3E-12
	***msrA***-Peptide methionine sulfoxide reductase (*Y. pseudotuberculosis*, YPTS_0481)	62/79	179	5E-37
	***resP***-Putative resistant protein (*Enterobacter nickellidurans*, AM003901)	86/95	129	1E-15
	***surA***-Peptidyl-prolyl cis-trans isomerase (*P. luminescens*, plu0611)	81/93	182	7E-21
	***uspB***-Universal stress protein (*P. luminescens*, plu0120)	90/100	100	5E-11
	***xkIS***-Putative transposase (*Nitrosococcus oceani*, Noc_1882)	54/66	360	9E-30
	***rep***-Putative rep protein (*Salmonella enteritidis*, SeSA_C0001)	77/80	192	3E-21
	***res***-Putative type III restriction enzyme, res subunit (*Bacillus coagulans*, BcoaDRAFT_2435)	72/87	261	2E-31
	***rpoB***-DNA-directed RNA polymerase (*Y. pseudotuberculosis*, YPTS_0304)	98/100	240	3E-45
	***rrmB***-16S ribosomal RNA methyltransferase (*P. luminescens*, plu4252)	88/94	150	3E-18
**Metabolism**				
- Amino acid synthesis	***lysC***-Aspartate kinase (*S. proteamaculans*, Spro_4479)	88/93	182	2E-30
- Cofactor biosynthesis	***ubiE***-Ubiquinone biosynthesis O-methyltransferase (*S. proteamaculans*, Spro_3270)	100/100	107	8E-17
- Cofactor metabolism	***cobJ***-Precorrin-3b c17-methyltransferase (*Escherichia blattae*, REB001118)	83/91	210	2E-27
- Glyoxylate pathway	***aceE***-Pyruvate dehydrogenase E1 component (*P. luminescens*, plu3623)	92/96	156	2E-20
	***aceK***-Bifunctional isocitrate dehydrogenase kinase/phosphatase protein (*P. luminescens*, plu4394)	88/95	174	2E-22
	***dld***-D-lactate dehydrogenase (*P. luminescens*, plu2848)	83/92	251	4E-37
- Lipid synthesis	***atoB***-beta-ketoadipyl CoA thiolase (*Escherichia fergusonii*, EFER_1599)	100/100	99	2E-05
- Nucleotide synthesis	***purF***-Amidophosphoribosyltransferase (*P. luminescens*, plu3167)	97/97	111	7E-13
- Protein degradation	***clpP***-ATP-dependent Clp protease proteolytic subunit (*Y. enterocolitica*, YE3134)	76/88	266	6E-45
- Protein folding	***trxB***-Predicted redox protein, regulator of disulfide bond formation (*Yersinia pestis*, YP_3227)	88/90	123	2E-12
**Nutrition**				
- Ion uptake	***znuA***-ABC high-affinity zinc uptake transporter, periplasmic binding protein (*P. luminescens*, plu2115)	81/87	141	2E-13
- Sugar uptake	***malF***-Maltose ABC transporter, permease protein (*P. luminescens*, plu0459)	88/93	168	3E-22
**Unknown**				
	***xkst1***-Hypothetical protein (*Ruminococcus gnavus*, RUMGNA_01513)	79/87	141	9E-14
	***xkst2***-Hypothetical protein (*P. luminescens*, plu2317)	76/87	162	8E-9
	***xkst3***-Hypothetical protein (*P. luminescens*, plu3301)	84/93	165	4E-20
	***xkst4***-No similarity to known genes			
	***xkst5***-No similarity to known genes			
	***xkst6***-No similarity to known genes			

Underlined genes were also upregulated in *Photorhabdus temperata *during infection of *R. majalis*. Other genes were exclusively induced in *X. koppenhoeferi *and similar sequences of double-underlined genes were absent in the genome of *P. temperata*.

### Identification of Photorhabdus temperata or Xenorhabdus koppenhoeferi specific genes

**Figure 4:**
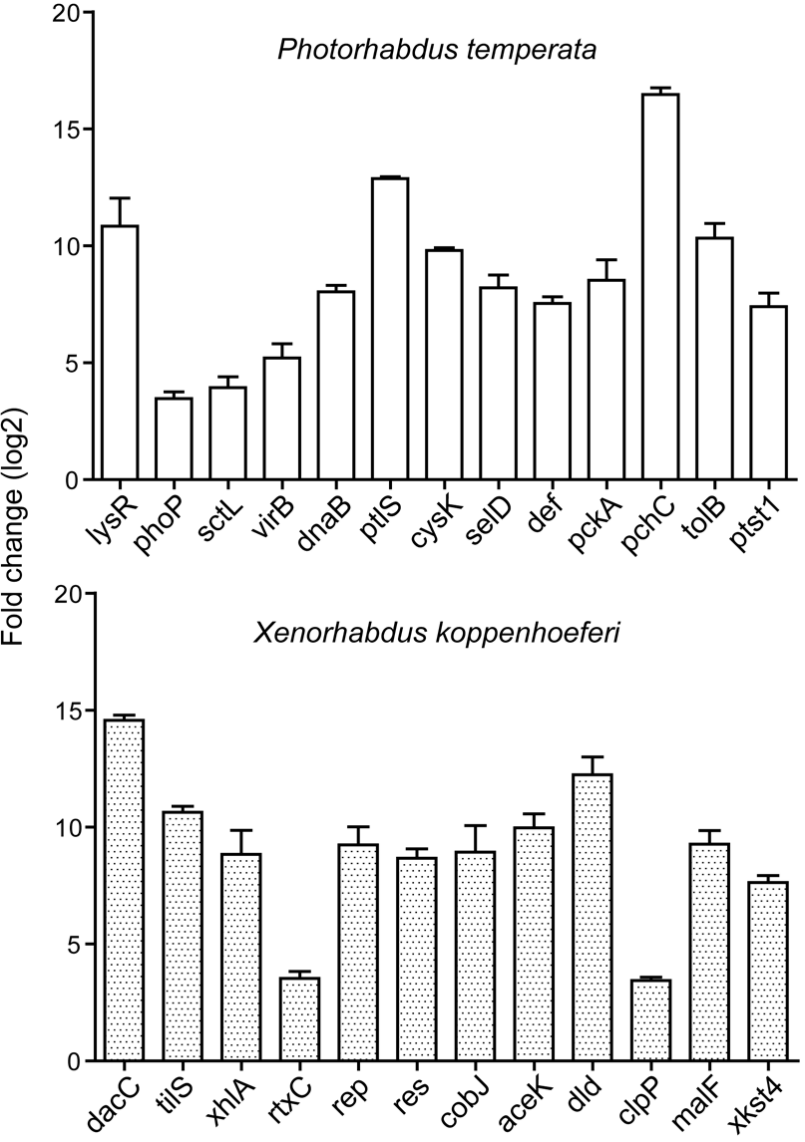
**Quantitative real time PCR results showing fold changes in the expression of selected *Photorhabdus temperata *and *Xenorhabdus koppenhoeferi *genes identified by SCOTS upon infection of the insect host *Rhizotrogus majalis***.

To identify genes that were distinctive in *P. temperata *or *X. koppenhoeferi *genome or exclusively induced in one or the other bacterium, comparative hybridization was performed based upon the identified genes. Twenty nine of the 40 distinct *P. temperata *transcripts have similar sequences in *X. koppenhoeferi *genome, thus 11 were *P. temperata *specific; 30 of the 39 distinct *X. koppenhoeferi *transcripts share sequence similarity with *P. temperata *genome, thus 9 were specific to *X. koppenhoeferi *(Tables 1 and 2). Although these 59 transcripts (29 + 30) corresponding to genes present in the genome of both bacteria, 21 from *P. temperata *and 22 from *X. koppenhoeferi *were identified to be exclusively induced in each of them. Only a small number of transcripts were induced in both bacteria, and most of them corresponded to genes required for stress response. Further, the level of changes in the transcripts exclusively induced in one or the other bacterium during infection of *R. majalis *was determined by quantitative real-time PCR (qRT-PCR) on following representative genes: *cysK*, *def*, *dnaB*, *lysR*, *pchC*, *pckA*, *phoP*, *ptst1*, *sctL*, *selD*, *tolB *and *virB *from *P. temperata*, and *aceK*, *clpP*, *cobJ*, *dacC*, *dld*, *malF*, *res*, *rtxC*, *tilS*, *virH*, *xhlA*, and *xkst4 *from *X. koppenhoeferi*. qRT-PCR results displayed consistence with the SCOTS assay. Most tested genes displayed 6-16 fold induction in qRT-PCR assays, and a few genes including *sctL*, *phoP*, *rtxC *and *clpP *exhibited about 3-fold induction (Fig. 4).

### Analysis of biological associations of in vivo-induced genes

To understand the function and interactions of the identified gene products, interactive networks were built to provide an overview of how gene products relate to each other by leveraging databases of published literature. Using PathwayStudio (Ariadne, Rockville, MD, USA) program, we were able to distill all published information about biological relationships of bacterial homologs of the identified genes. Molecular interactions linking each identified gene product in terms of binding, direct molecular regulation, effects on expression, transportation, and molecule synthesis were elucidated in PathwayStudio. Based on this analysis, the biological significance of each gene was inferred. Linkage between proteins shows how upregulated genes may influence other genes or gene products. For example, gene *rpoB*, induced in both bacteria, interacts with many gene products, suggesting its overall importance during insect infection (see Additional files 1 and 2). Other identified genes with multiple connections to different gene products were different between *P. temperata *and *X. koppenhoeferi*, indicating distinct biological processes used by the two bacteria for insect infection. Moreover, linkages with small molecules indicate how normal functions in gene regulation networks are influenced by small molecules that may be available in the insect body. For example, regulation of genes *yncB *and *purH *in *P. temperata *are influenced by aromatic compounds and formate, respectively (see Additional file 1). In *X. koppenhoeferi*, gene *trxB *is influenced by arabinose and selenium, and gene *znuA *is influenced by etoposide (see Additional file 2). Compared to the biological interactions (see Additional files 1 and 2), molecular pathways or networks in which identified genes link to each other were further illustrated (see Additional file 3). In total, 20 genes from *P. temperata *and 18 from *X. koppenhoeferi *(see Additional file 3) were found to be part of the dense protein interaction networks, indicating that these genes may act in concert to achieve successful infection. Some products encoded by induced genes were found to interact with as many as eight other gene products in the network. For example, *P. temperata pckA *and *X. koppenhoeferi aceE *both influence 8 other genes in the network (see Additional file 3). As a whole, the protein networks of the identified genes were different for the two bacteria except that NAD synthetase gene *nadE *was found to be common to both bacteria.

## Discussion

**Figure 5:**
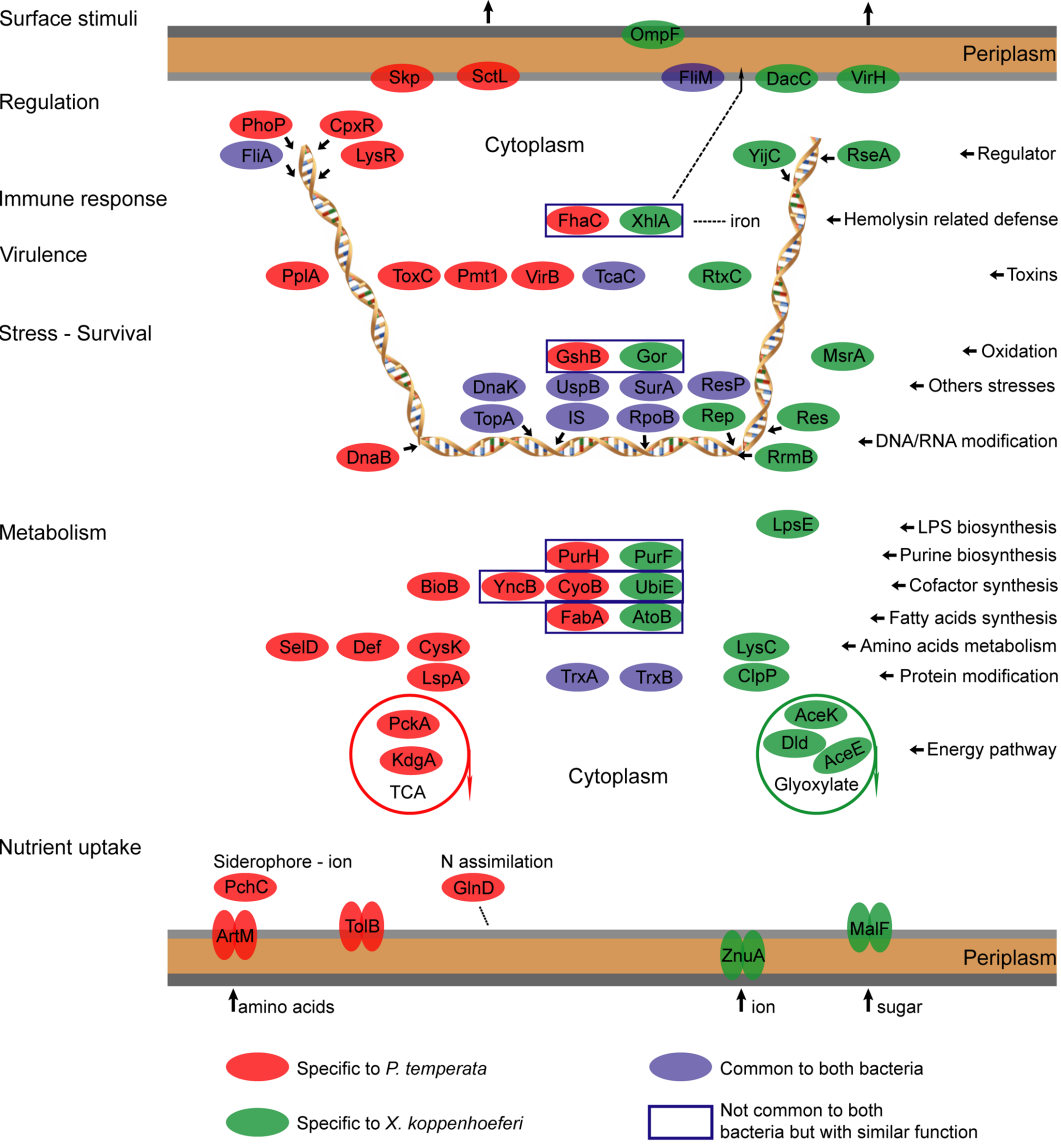
**Conceptual molecular model illustrating comparative contributions of the SCOTS identified genes in bacteria *Photorhabdus temperata *and *Xenorhabdus koppenhoeferi *during infection of the insect *Rhizotrogus majalis***. The set of common and different genes revealed similar and different molecular mechanisms of pathogenicity in *P. temperata *and *X. koppenhoeferi *during infection of *R. majalis*. The common gene products were defined by either sequence similarity or similar functions. The detailed possible functions for individual gene products are listed in Tables 1 and 2.

Our gene expression data reveal similarities and differences in molecular mechanisms of pathogenicity by the two bacteria with apparently similar biology. As depicted in the conceptual molecular model (Fig. 5), apart from a relatively small number of common strategies used by *P. temperata *and *X. koppenhoeferi *to cause infection of *R. majalis*, most upregulated genes were different between the two bacteria, suggesting vastly different pathways to bring about infection of the same insect host.

### Genes involved in cell structure modification

As the physical contact between bacteria and their host is accomplished by the outer surface, the bacterial cell envelope is crucial for communication and interaction with host cells during infection. Genes involved in the synthesis of outer membrane proteins (*skp *in *P. temperata *and *ompF *in *X. koppenhoeferi*) and flagella (*fliA *in *P. temperata *and *fliM *in *X. koppenhoeferi*) were induced in both bacteria, indicating the importance of surface proteins in this bacteria-insect interface. Outer membrane proteins may contribute to the modulation of bacterial cell surface properties during interaction with the host, thus playing a role in evading host immune response [18]. Flagellum-mediated motility may provide an advantage for infection by attachment to the host surface [19,20], whereas it has been reported in *X. nematophila *that the expression of virulence factors is not controlled by the regulation of flagella motility [21]. Gene *dacC *encoding a peptidoglycan synthesis enzyme D-alanyl-D-alanine carboxypeptidase was induced in *X. koppenhoeferi *but not in *P. temperata*, implying that peptidoglycan undergoes structural alterations in *X. koppenhoeferi *that probably add to its fitness when residing in the host. As a well-defined virulence factor playing a major role in the development of septicemia [22], lipopolysaccharide biosynthesis gene *lpsE *was induced in *X. koppenhoeferi*, but not in *P. temperata*, which may explain in part why relatively slower growing *X. koppenhoeferi *did not differ from *P. temperata *in virulence to *R. majalis *in our previous studies [17].

### Genes involved in regulatory networks

Facing stimuli, different regulatory genes were upregulated in *P. temperata *and *X. koppenhoeferi*, reflecting their unique ability to respond to environmental challenges. *phoP *and *cpxR *genes identified from *P. temperata*, encode response regulators in two-component regulatory systems PhoPQ and CpxRA, respectively [23,24]. PhoP and CpxR are dual transcriptional regulators that are activated in response to environmental stimuli and then act to modulate activity of other genes [23,24]. Responding to low Mg^2+ ^levels, *phoP *expression is auto-regulated by PhoP and PhoQ proteins and *phoQ *is constitutive [25]. Auto-regulation of *phoP *likely regulates expression of other genes that mediate various cellular functions such as LPS modification, cell structure [26], and type III secretion systems [27]. *phoP *has been previously identified to be involved in virulence as mutational inactivation of this gene rendered *P. luminescens *avirulent to *Spodoptera littoralis *larvae [6]. Similarly, the regulatory protein CpxR can be activated in response to signals associated with growth and metabolic pathways [28]. By sensing cell envelope stress CpxR appears to control expression of other genes involved in envelope stress response, secretion, motility and multidrug resistance [29]. Sometimes, CpxR acts to modulate other transcriptional factors [30]. Although there is no evidence showing links between *phoP*/*cpxR *and other isolated genes, overexpression of these two response regulator genes may possibly relate to genes involved in stress responses (*gshB*, *gor*, *msrA *and *surA*), cell structure (*skp*, *dacC *and *ompF*) and type III secretion systems (*sctL*) identified in this study. Further, in *P. temperata*, *lysR *gene encoding a transcriptional activator protein was found to be upregulated during infection of *R. majalis*, while two genes *yijC *and *rseA *encoding transcriptional repressors were identified in *X. koppenhoeferi*. The pathway network analysis indicates that the product of *yijC *may function as a repressor to potentially control the expression of the *fabB *gene, which in turn, modulates the physical properties of the membrane by altering the level of unsaturated fatty acid production. Besides transcriptional regulatory genes, gene *tilS*, encoding a putative cell cycle protein [4], was induced in *X. koppenhoeferi*. Upregulation of distinct regulatory genes in these two bacteria indicates the different stress response mechanisms between the bacterial species in the same host. However, none of these regulatory factors were identified in *P. luminescens *exposed to *G. mellonella *homogenate in a previous study [16], suggesting that regulation of these factors either depends on host (*R. majalis *not *G. mellonella*) or bacteria themselves.

### Genes involved in virulence and secretion

Identification of genes encoding hemolysin related proteins in *P. temperata *(*fhaC*) and *X. koppenhoeferi *(*xhlA*) may suggest their role in dealing with the insect cellular immune response and iron scavenge [31,32]. Subsequently, bacterial virulence factors actively contribute to a successful infection by colonization of and toxicity towards the insect host. Among the virulence genes identified in this study, *tcaC *was identified to be induced in both *P. temperata *and *X. koppenhoeferi *during infection of *R. majalis*. The *tca *gene family encodes four toxin complexes TcaA, TcaB, TcaC and TcaD [33], and has been found in several bacterial pathogens including *P. luminescens*, *Yersinia *and *Xenorhabdus *[34]. Interestingly, Tca toxin encoding genes were also found to be induced in a related species *P. luminescens *treated with the homogenate of *G. mellonella *[16]. This leads us to speculate that TcaC serves as either an activator or a chaperone in secretion of toxin complex from the cell as proposed by ffrench-Constant et al. [35] and Waterfield et al. [36]. Induction of gene *pplA*, encoding a phospholipase, in *P. temperata *is expected to contribute to its faster growth rate [37] compared to *X. koppenhoeferi *in *R. majalis*. Further, genes encoding components of different types of secretion machineries were isolated in this study. Upregulation of gene *sctL*, encoding components of type III secretion machinery, possibly coordinates protein secretion system in *P. temperata *for translocating effector molecules across the bacterial cell envelope into host cells [38-40]. Unlike *Photorhabdus*, the genomes of *Xenorhabdus bovienii *and *X. nematophila *do not encode homologues of a dedicated type III secretion system [3], indicating that *Xenorhabdus *likely uses different effectors or secretion systems. Notably, in *X. koppenhoeferi*, *virH *with sequence similarity to a gene encoding putative toxin secretion transporter in *Vibrio cholera*, was upregulated during infection of *R. majalis*. In addition, several other genes encoding proteins with putative virulence functions identified in this study may represent novel toxin candidates involved in insect infection. These include *pmt1*, *toxC *and *virB *from *P. temperata*, and *virH *from *X. koppenhoeferi*. Differences in expression of virulence and secretion related genes imply the differential virulence mechanisms employed by the two bacteria.

### Genes involved in adaptation to host-induced stresses

Compared to all other functional classes, more stress response genes were identified to be shared by *P. temperata *and *X. koppenhoeferi*, suggesting common themes in adaptation to host-imposed stresses. Upregulation of both *surA *and *uspB *in both bacteria indicates their importance in bacterial adaptation to global stress during insect infection. Gene *surA *is important for bacterial survival during infection [41,42] to avoid accumulation of non-native protein conformations [42-44], and *uspB *gene product is usually accumulated in the stationary phase and probably important for ethanol stress [45]. Similarly, induction of a chaperone protein encoding gene *dnaK *in both bacteria may picture common stress responses between *P. temperata *and *X. koppenhoeferi *in a number of cellular processes including rescue of misfolded proteins and control of the activity of folded regulatory proteins [46]. The glutathione synthetase gene *gshB *which mediates condensation of gamma-glutamylcysteine and glycine to form glutathione [47,48] was induced in *P. temperata*, but glutathione reductase gene *gor *which reduces glutathione disulfide to the sulfhydryl form of glutathione [47,48] was induced in *X. koppenhoeferi*. Glutathione plays a major role in protection against oxidative stress and in detoxification of hazardous chemicals or heavy metals [49]. Thus, although *gshB *and *gor *corresponding products are involved in different glutathione biosynthesis pathways, expression of these two genes may suggests possible role of detoxification mechanism in *P. temperata *and *X. koppenhoeferi *during infection of *R. majalis*. Further, as *Photorhabdus *and *Xenorhabdus *bacteria can switch their phenotypes upon insect infection, we speculate that upregulation of transposase genes in both bacteria may contribute to genetic variability for better adaptation and survival in a particular niche [50]. Apart from the common mechanisms handling stresses, gene *msrA *encoding a peptide methionine sulfoxide reductase was unique to *X. koppenhoeferi*. As an important antioxidant enzyme, peptide methionine sulfoxide reductase mediates the repair of proteins damaged by sulfoxidation of methionine residues [51,52]. Products of *msrA *are found to interact with several other gene products (see Additional file 2), also suggesting its importance during insect infection.

### Genes involved in intracellular metabolism

Induction of different metabolism genes in *Photorhabdus *and *Xenorhabdus *during insect infection highlights the differences in metabolic changes between the two bacteria. Three genes, *aceE*, *aceK *and *dld*, whose products catalyze subsequent steps of the glyoxylate pathway [53], were induced in *X. koppenhoeferi*. In contrast, two genes *kdgK *and *pckA *involved in energy metabolism in the TCA cycle were upregulated in *P. temperata *[54,55]. Genes related to the TCA cycle have been also found to be induced in other bacteria upon host infection, for example in *P. luminescens *in the presence of *G. mellonella *homogenate [16] and in *Vibrio cholerae *upon infection of infant mice [56,57]. Further, as a cofactor in the metabolism of fatty acids and leucine, biotin plays a role in TCA cycle by assisting in metabolic reactions and helping to transfer carbon dioxide [58]. Therefore, upregulation of *bioB *encoding a biontin synthetase protein further suggests that TCA cycle is involved in *P. temperata*. Induction of different amino acid biosynthetic genes may suggest the differences in amino acid requirements in the two bacteria during infection. Induction of *cysK *gene in *P. temperata *suggests that cysteine may be required when growing in the insect, but lysine may be needed for *X. koppenhoeferi *as *lysC *gene was induced. Isolation of *P. temperata def*, encoding an amino acid tRNA synthesis enzyme N-formylmethionylaminoacyl-tRNA deformylase, may reflect the importance of specific amino acids involved in the translation process during growth in the insect host. Indeed, the pathway analysis reveals that *def *gene product interacts with CysS, a cysteinyl-tRNA synthetase (see Additional file 1), also suggesting the requirement for cysteine in *P. temperata *during infection. Genes involved in protein synthesis and degradation can also be distinguished between the two bacteria. A protease gene, *clpP*, involved in protein degradation, was induced in *X. koppenhoeferi *upon infection. In fact proteases have been identified as virulence factors in some other bacterial pathogens [59]. The signal peptidase II encoded by *lspA *gene induced in *P. temperata *belongs to a class of aspartyl proteases. Since the product of *lspA *has been suggested as an important virulence factor operating via maturation of several lipoproteins [60], we postulate that the product of *lspA *may play a pathological role in *P. temperata *by cleaving some core proteins and thus influencing bacterial life cycle in the insect host.

### Genes involved in nutrient scavenging

Induction of different types of nutrient uptake systems in *Photorhabdus *and *Xenorhabdus *further emphasized the possible differences in nutritional requirements of the two bacteria in the insect host. Ion scavenging systems induced in *P. temperata *and *X. koppenhoeferi *are completely different. In *P. temperata*, *pchC*, a gene involved in the biogenesis of pyochelin type of siderophore [61], was upregulated indicating the importance of iron, whereas a siderophore-independent zinc ion transport system encoded by gene *znuA *[62] was induced in *X. koppenhoeferi *indicating the importance of zinc. Also supported by the pathway analysis, product of *glnD *identified from *P. temperata *likely controls the production of siderophore (see Additional file 1). In addition to ion scavenging systems, *artM*, encoding a putative ABC amino acid transport system [63] was identified in *P. temperata*, but not in *X. koppenhoeferi*. Besides transporting L-arginine across the inner membrane [63], the pathway network reveals that the product of *artM *participates in cystine transport (see Additional file 1), suggesting that this gene may also mediate cystine amino acid acquisition. Upregulation of *artM *in *P. temperata *also suggests that amino acids may be available for uptake in the insect. Similarly, it has been discussed previously that histidine and phosphatidylethanolamine, the possible components presented in the insect host hemolymph, may be important food sources used by *P. luminescens *for growth within the insect host [64]. As cysteine synthesis gene *cysK *was also identified from *P. temperata *as discussed above, it may indicate that the level of cysteine either synthesized by *P. temperata *or acquired from the insect host is not sufficient during infection. Interestingly, *X. koppenhoeferi *seems to require sugar as *malF *gene which is involved in uptake for maltose and lactose was upregulated. Further, *P. temperata *but not *X. koppenhoeferi *induced *tolB *gene that encodes a Tol protein upon insect infection. As a periplasmic protein, TolB is an extra member in the Tol system. In *E. coli*, Tol system stabilizes the outer membrane structure and mutations in Tol encoding genes result in hypersensitivity to deleterious agents [65]. Despite unknown mechanistic details of Tol system, upregulation of *P. temperata tolB *gene suggests that it may be involved in importing biomolecules by somehow supplying energy or transport as noted by Nikaido [66] in other bacteria.

### Genes with unknown function

Ten genes encoding products with unknown functions were identified to be upregulated in both bacteria during infection of *R. majalis*. These include two *P. temperata *and three *X. koppenhoeferi *genes which show sequence similarity to genes encoding hypothetical proteins with unknown functions, and two *P. temperata *and three *X. koppenhoeferi *sequences that exhibit no similarity to any gene or gene product in current databases. These unknown genes may represent novel pathogenicity factors used by bacteria during insect infection and thus require further investigation.

## Conclusion

This research indicates that selective capture of transcribed sequences (SCOTS) is a powerful technique to study *in vivo *gene expression in bacterial pathogens of insects. Results reveal that phylogenetically related bacteria with similar biology differ dramatically in molecular mechanisms of pathogenicity in the same host. *Photorhabdus *and *Xenorhabdus *bacteria appear to share more genes in host-induced stress protection category, including tolerance against universal stresses, glutathione-mediated protection against oxidative stress, and protection against nucleic acid damage, compared to genes in all other categories. While genes encoding TcaC toxin and hemolysin related proteins were identified to be induced in both *P. temperata *and *X. koppenhoeferi*, the two bacteria seem to use different regulatory genes, secretion systems and virulence factors to achieve infection of the same insect host *R. majalis*. Further, *P. temperata *and *X. koppenhoeferi *displayed different metabolic adaptations in the same insect host as reflected by the high proportion of identified genes involved in metabolism. Overall, this study provides a better understanding of the evolution of bacterial pathogenesis and provides molecular targets for genetic manipulation of these economically important nematode symbiotic insect pathogens.

## Methods

### Bacteria, insects, and culture conditions

The symbiotic bacteria *P. temperata *strain GPS11 [17] and *X. koppenhoeferi *strain USNJ01 [67] were isolated from nematodes *H. bacteriophora *strain GPS11 [68] and *S. scarabaei *strain AMK001 [69], respectively. The *H. bacteriophora *GPS11 were obtained from our liquid nitrogen frozen stock, and of *S. scarabaei *AMK001 were obtained from Dr. Albrecht M. Koppenhöfer (Rutgers University, New Brunswick, New Jersey). The bacteria were cultured in BHI broth at 25°C unless otherwise stated. The second instar larvae of the white grub *R. majalis *were collected from Sunleaf Nursery (Madison, Ohio). The field collected insect larvae were kept at room temperature for 10 days, and only healthy, actively moving larvae were used in all experiments.

### General techniques

Bacterial genomic DNA was prepared using standard method for Gram negative bacteria [70]. Biotinylation of bacterial genomic DNA was obtained with EZ-Link Psoralen-PEO-Biotin (Pierce) according to the manufacturer's instructions. The total RNA was isolated using TRIzol reagent (Invitrogen) according to the manufacturer's instructions. RNA samples were treated with RNase-free DNase I (Ambion, Austin, TX) according to the manufacturer's guidelines, and were checked by spectrophotometer and gel electrophoresis. Total RNA was converted to first-strand cDNA using Superscript II reverse transcriptase (Invitrogen RT-PCR kit) according to the manufacturer's instructions. First strand cDNA was made double-strand with Klenow fragment (NEB, Beverly, MA) as described by Froussard [71].

### Isolation of bacterial transcripts induced in Rhizotrogus majalis

SCOTS technique was used to identify *in vivo *gene expression of *P. temperata *and *X. koppenhoeferi *during infection of *R. majalis*. For the control, three 5 μg total RNA samples isolated from 48 h bacterial cultures were used to make double-strand cDNAs using the random primer SCOT09 (5-ATCCACCTATCCCAGTAGGAGNNNNNNNNN). The synthesized cDNAs were PCR amplified using the primer SCOT0 (5-ATCCACCTATCCCAGTAGGAG) for 30 cycles. For the treatment, 10 μl suspension of 48 h *P. temperata *or *X. koppenhoeferi *cultures containing 1 × 10^4 ^cells was injected into the hemolymph of *R. majalis *from the base of the foreleg and incubated at 25°C. We evaluated bacterial colonization and found that bacteria of *P. temperata *and *X. koppenhoeferi *were 5 × 10^5 ^and 2 × 10^5 ^colony-forming units (CFU) per insect, respectively, at 24 h post injection, suggesting development of septicemia. Total RNA was isolated from the whole insects at 24 h post injection when the insects were still alive (The insects died within 24 - 48 h). Three 5 μg RNA samples obtained from three infected insects for each bacterium were used to made double-strand by the random primer SCOT189 (5-GACAGATTCGCACTTAACCCTNNNNNNNNN). Double-strand cDNAs were then PCR amplified for 30 cycles by primer SCOT18 (5-GACAGATTCGCACTTAACCCT). Bacterial cDNA normalization was done as described below. In this study, the ribosomal operons (rRNA) of *P. temperata *and *X. koppenhoeferi *were amplified and used to block the abundant rRNA sequence in order to effectively capture the cDNA molecules representing mRNA transcripts. The rRNA operon was added to biotinylated genomic DNA at a ratio of 10:1. The genomic DNA-rRNA mixture was sonicated to a size range of 1 to 5 kb. The sonicated, biotinylated genomic DNA-rRNA mixture containing 6 μg rRNA and 0.6 μg genomic DNA was denatured and hybridized for 30 min at 65°C. PCR amplified cDNAs (6 μg) were denatured and added to the genomic DNA-rRNA prehybridized mixture, and hybridized at 65°C for 24 h. Streptavidin magnesphere paramagnetic particles (Invitrogen) were used to capture the bacterial cDNAs that hybridized to biotinylated genomic DNA according to the manufacturer's instructions. Captured cDNAs were then eluted, precipitated, and amplified by PCR with the primer SCOT0 for additional two successive rounds of SCOTS. After three rounds of SCOTS, the three amplified cDNA samples for each bacterium were pooled, and the pooled cDNA mixtures were considered to be normalized *in vitro *or *in vivo *cDNA library.

To enrich the normalized *in vivo *cDNAs, the normalized *in vivo *cDNAs from each bacterium were hybridized to biotinylated genomic DNA that has been prehybridized with both rRNA and normalized *in vitro *cDNAs. After hybridization, the bacterial cDNAs were captured and PCR amplified for next round of enrichment. Finally, the amplified enriched bacterial cDNAs were cloned into an original TA cloning vector (Invitrogen) to construct the enriched *in vivo *cDNA library.

### Southern blot screening of SCOTS enriched transcripts

Inserts from the enriched *in vivo *cDNA library were screened to confirm to be upregulated by southern blot hybridization which has been used as a confirmatory test in most other SCOTS-based studies [72-76]. Individual clones from each subtractive library were randomly picked and amplified by PCR using M13 primers. Ten micro liter of each amplified product was mixed with 70 μl 20 × SSC (1 × SSC is 0.15 M NaCl plus 0.015 M sodium citrate), and then 80 μl sample mixture was transferred to each well of dot-blot containing the positively charged nylon membrane at the low vacuum. The nylon membrane with samples was denatured with denature buffer (3 M NaCl, 0.4 M NaOH) for 10 min at room temperature, and neutralized in 1 × PBS buffer (0.1 M NaCl, 7 mM Na_2_HPO_4_, and 3 mM NaH_2_PO_4_, pH 6.8) for 10 min at room temperature. The membrane was dried by baking at 80°C for 2 h, and then the baked membrane was rinsed with 2 × SSC and soaked in 2 × SSC for 5 min. The membrane was transferred into a hybridization bottle for hybridization using Dig easy hyb granules (Roche) according to the manufacturer's instruction. Normalized, insect-derived and broth-derived cDNA pools were labeled using the PCR DIG Probe Synthesis kit (Roche) for use as probes. The probes were denatured and added to the hybridization bottles containing the membrane and the hybridization buffer. Hybridization continued at 65°C for approximately 24 h. The membrane was washed briefly with 2 × SSC at room temperature and then twice with 1 × SSC - 0.1% SDS for 15 min each time; these washes were done at 65°C. A final brief rinse with 0.1 × SSC at room temperature completed the washing process. The membrane was incubated at room temperature with 4 ml 1 × SSC with 8% dry milk for 30 m. Four milliliter dilution of anti-digoxigenin-HRP conjugate (1: 800) was added, and incubation continues 1 h at room temperature. The membrane was briefly washed as describe above, and successful hybridization was detected by Amersham ECL Plus western blotting detection reagents (GE healthcare Bio-Sciences Corp, Piscataway, NJ) using chemiluminescent detection. The individual clones that only hybridized to the probe made from normalized *in vivo *cDNAs were chosen for sequence analysis.

### Analysis of bacterial genes induced in Rhizotrogus majalis

The samples were sequenced at the Biotechnology Center, Madison, WI, USA. These sequences were edited and assembled using Editseq (DNASTAR) and Contig (Vector NTI). Similar sequences were identified using BLAST algorithms (blastx and tblastx) in GenBank of National Center for Biotechnology Information  and database of *Xenorhabdus *genomes (. The functions of identified sequences were assigned using Gene Ontology . The redundancy of each gene was counted cumulatively and the library coverage was calculated using Analytic Rarefaction ([77] which has been used for estimation of total transcript diversity in the cDNA library [78-81].

### Validation of SCOTS procedure

Semi-quantitative reverse transcriptase PCR (RT-PCR) was performed as described previously [82] to verify results obtained by SCOTS analysis. To confirm that the identified genes were differentially induced during infection of the insect hemolymph compared to the culture, RNA was prepared from either infected insect or broth cultured log-phase bacterial cells and DNAse treated. RT-PCR was carried out using 5 μg of RNA in the presence of 5 pmol of gene specific reverse primers and the primer targeting 16S rRNA gene for an internal positive control together in the same reaction. The cDNA template was then diluted 1:100 with sterile ddH_2_O for use in subsequent PCR. Twenty-five cycles of PCR were performed using primer pairs for specific genes and 16S rRNA gene in separate vials, but from the same master mix, using 5 μl of diluted cDNA as template. In addition, a eukaryotic housekeeping gene 18S rRNA was used as a control to evaluate if bacterial cDNAs were purified apart from the insect cDNAs after SCOTS normalization. For this purpose, presence of 18S rRNA gene in the *in vivo *cDNA populations before and after SCOTS normalization was measured by PCR amplification using 50 ng cDNA samples and primers 18SF (5-GGAATTGACGGAAGGGCACCA) and 18SR (5-CCAGACAAATCGCTCCACCAAC). Also, a prokaryotic housekeeping gene gyrase A (*gyrA*) was used as another control to validate that only differentially expressed genes were captured after enrichment. The presence of *gyrA *in cDNA populations of enriched *in vivo *cDNAs, and *in vitro *and *in vivo *cDNAs before and after SCOTS normalization was evaluated by PCR using primers gyrAF (5-ACGCGACGGTGTACCGGCTT) and gyrAR (5-GCCAGAGAAATCACCCCGGTC). All experiments were performed at least three times.

### Comparative hybridization and quantitative real-time PCR

Comparative hybridization was performed to assess exclusivity of the identified genes to *P. temperata *or *X. koppenhoeferi*. This approach has been validated for cross-species comparisons of gene expression in many studies [83-86]. To determine the genomic presence of the identified genes, genes identified from one bacterial species were individually screened by cross-hybridization to the sonicated biotinylated genomic DNA of the other, followed by PCR amplification of the eluted hybrids using the defined primers SCOT18. The genes with sequence present in both bacterial genomes were singled out to further evaluate their induction specificity to *P. temperata *or *X. koppenhoeferi*. The selected genes induced in one bacterial species were individually screened by cross-hybridization to a heterologous probe made by pre-hybridization of sonicated biotinylated genomic DNA with the respective rRNA operon and normalized *in vitro *cDNAs of the other, and subsequently amplified using the defined primer SCOT18. Quantitative real-time PCR (qRT-PCR) was further used to validate and quantify the change of genes identified exclusively in one or the other bacterium, excluding genes without sequence similarity between the two bacteria but with similar functions. These representative genes were chosen for analysis based on the availability of designing qRT-PCR primers using identified sequences. Total RNA was prepared from bacteria grown *in intro *or *in vivo *as described above. Real-time PCR was performed in an IQ5 machine (Bio-Rad) using QuantiTect SybrGreen PCR Kit (Qiagen) according to the manufacturer's instructions. The specific gene primers for real-time PCR were listed in the Additional file 4. All reactions were run in triplicate with three independent cDNA samples and a no cDNA template negative control. According to a previous study on *Photorhabdus *bacteria [87], 16S rRNA gene was chosen as an internal control for normalization. The calculated threshold cycle (Ct) was normalized to the Ct of the 16s rRNA gene amplified from the corresponding sample, and the relative change in expression of a gene *in vivo *as compared to *in vitro *was calculated as previously described [88].

### Pathway analysis

To depict biological interactions influenced by identified genes, we built information rich predicted interaction networks using the ResnetCore bacterial molecular database of the PathwayStudio 5.0 (Ariadne, Rockville, MD, USA). PathwayStudio is a program for visualization and analysis of biological pathways and gene regulation networks. This program comes with the ResNet database of more than 500,000 functional relationships and the MedScan tool for automatic extraction of information from the scientific literature. First, the direct interaction with proteins and small molecules for each identified gene was visualized using PathwayStudio. Then, the interaction network of identified genes was built based on relationships between gene products or small molecules in form of binding, direct molecular regulation, effects on expression, molecule transport, protein modification, and molecule synthesis.

## List of abbreviations

IJs: infective juveniles; SCOTS: selective capture of transcribed sequences.

## Authors' contributions

RA performed all the experiments, analyzed the data, wrote the first draft, and participated in the design of the study. SS helped in the design of the study and provided comments on the manuscript. PSG obtained the funding for the research, designed the study, and helped in writing of the paper. All authors read and approved the final manuscript.

## Supplementary Material

Additional file 1 **Direct linkages of proteins and small molecules to the SCOTS identified *Photorhabdus temperata *genes induced upon infection of *Rhizotrogus majalis***. Linkages of molecules to identified genes were built in the PathwayStudio program. Gray and green ovals indicate small molecules. The gene products are represented by red or blue ovals, where blue ovals indicate genes identified in this study and red ovals represent genes in the database of the PathwayStudio program. The relationships are indicated by lines as follows: Binding - violet links with violet circles, MolTransport - gray arrows with green rectangles, MolSynthesis - blue arrows with blue rectangles, ProtModification - brown arrows, Regulation - gray links with gray rectangles, PromoterBinding - green arrows with green circles, and Expression - blue arrows with blue rectangles. Arrows with "+" indicate positive regulation and with "-" indicate negative regulation. Click here for file

Additional file 2 **Direct linkages of proteins and small molecules to the SCOTS identified *Xenorhabdus koppenhoeferi *genes induced upon infection of *Rhizotrogus majalis***. Linkages of molecules to identified genes were built in the PathwayStudio program. Gray and green ovals indicate small molecules. The gene products are represented by red or blue ovals, where blue ovals indicate genes identified in this study and red ovals represent genes in the database of the PathwayStudio program. The relationships are indicated by lines as follows: Binding - violet links with violet circles, MolTransport - gray arrows with green rectangles, MolSynthesis - blue arrows with blue rectangles, ProtModification - brown arrows, Regulation - gray links with gray rectangles, PromoterBinding - green arrows with green circles, and Expression - blue arrows with blue rectangles. Arrows with "+" indicate positive regulation and with "-" indicate negative regulation. Click here for file

Additional file 3 **The network interactions between gene products of *Photorhabdus temperata *(panel A) and *Xenorhabdus koppenhoeferi *(panel B) built by leveraging databases of published literature**. The gene expression data obtained in this study were analyzed and visualized by PathwayStudio program. The gene products are represented by ovals, where blue ovals represent products encoded by genes induced in *P. temperata *(panel A) and *X. koppenhoeferi *(panel B) upon infection of the insect *Rhizotrogus majalis *and red ovals represent genes in the database of the PathwayStudio program. The product encoded by gene *nadE *was highlighted with a green circle as this gene was common to both bacteria and interacts with many other gene products in both networks. The relationships between gene products are indicated by lines as follows: Binding - violet links with violet circles, MolTransport - gray arrows with green rectangles, MolSynthesis - blue arrows with blue rectangles, ProtModification - brown arrows, Regulation - gray links with gray rectangles, PromoterBinding - green arrows with green circles, and Expression - blue arrows with blue rectangles. Arrows with "+" indicate positive regulation and with "-" indicate negative regulation. Click here for file

Additional file 4 **Supplementary Table 1**. Oligonucleotide sequences used for quantitative real-time PCR analyses. Click here for file

## References

[B1] Boemare N, Akhurst P (2006). The genera *Photorhabdus *and *Xenorhabdus*. Prokaryotes.

[B2] Forst S, Dowds B, Boemare N, Stackebrandt E (1997). *Xenorhabdus *and *Photorhabdus *spp.: bugs that kill bugs. Annu Rev Microbiol.

[B3] Goodrich-Blair H, Clarke DJ (2007). Mutualism and pathogenesis in *Xenorhabdus *and *Photorhabdus*: two roads to the same destination. Mol Microbiol.

[B4] Duchaud E, Rusniok C, Frangeul L, Buchrieser C, Givaudan A, Taourit S, Bocs S, Boursaux-Eude C, Chandler M, Charles JF, Dassa E, Derose R, Derzelle S, Freyssinet G, Gaudriault S, Medigue C, Lanois A, Powell K, Siguier P, Vincent R, Wingate V, Zouine M, Glaser P, Boemare N, Danchin A, Kunst F (2003). The genome sequence of the entomopathogenic bacterium *Photorhabdus luminescens*. Nat Biotechnol.

[B5] Waterfield NR, Daborn PJ, ffrench-Constant RH (2002). Genomic islands in *Photorhabdus*. Trends Microbiol.

[B6] Derzelle S, Turlin E, Duchaud E, Pages S, Kunst F, Givaudan A, Danchin A (2004). The PhoP-PhoQ two-component regulatory system of *Photorhabdus luminescens *is essential for virulence in insects. J Bacteriol.

[B7] Bennett HP, Clarke DJ (2005). The *pbgPE *operon in *Photorhabdus luminescens *is required for pathogenicity and symbiosis. J Bacteriol.

[B8] Krin E, Chakroun N, Turlin E, Givaudan A, Gaboriau F, Bonne I, Rousselle JC, Frangeul L, Lacroix C, Hullo MF, Marisa L, Danchin A, Derzelle S (2006). Pleiotropic role of quorum-sensing autoinducer 2 in *Photorhabdus luminescens*. Appl Environ Microbiol.

[B9] Eleftherianos I, Boundy S, Joyce SA, Aslam S, Marshall JW, Cox RJ, Simpson TJ, Clarke DJ, ffrench-Constant RH, Reynolds SE (2007). An antibiotic produced by an insect-pathogenic bacterium suppresses host defenses through phenoloxidase inhibition. PNAS.

[B10] Ji D, Kim Y (2004). An entomopathogenic bacterium, *Xenorhabdus nematophila*, inhibits the expression of an antibacterial peptide, cecropin, of the beet armyworm, *Spodoptera exigua*. J Insect Physiol.

[B11] Park Y, Herbert EE, Cowles CE, Cowles KN, Menard ML, Orchard SS, Goodrich-Blair H (2007). Clonal variation in *Xenorhabdus nematophila *virulence and suppression of *Manduca sexta *immunity. Cell Microbiol.

[B12] Cowles KN, Cowles CE, Richards GR, Martens EC, Goodrich-Blair H (2007). The global regulator Lrp contributes to mutualism, pathogenesis and phenotypic variation in the bacterium *Xenorhabdus nematophila*. Cell Microbiol.

[B13] Brugirard-Ricaud K, Givaudan A, Parkhill J, Boemare N, Kunst F, Zumbihl R, Duchaud E (2004). Variation in the effectors of the type III secretion system among *Photorhabdus *species as revealed by genomic analysis. J Bacteriol.

[B14] Brugirard-Ricaud K, Duchaud E, Givaudan A, Girard PA, Kunst F, Boemare N, Brehelin M, Zumbihl R (2005). Site-specific antiphagocytic function of the *Photorhabdus luminescens *type III secretion system during insect colonization. Cell Microbiol.

[B15] Park D, Forst S (2006). Co-regulation of motility, exoenzyme and antibiotic production by the EnvZ-OmpR-FlhDC-FliA pathway in *Xenorhabdus nematophila*. Mol Microbiol.

[B16] Münch A, Stingl L, Jung K, Heermann R (2008). *Photorhabdus luminescens *genes induced upon insect infection. BMC Genomics.

[B17] An R, Grewal PS (2007). Differences in the virulence of *Heterorhabditis bacteriophora *and *Steinernema scarabaei *to three white grub species: The relative contribution of the nematodes and their symbiotic bacteria. Biol Contr.

[B18] Rediers H, Rainey PB, Vanderleyden J, Mot RD (2005). Unraveling the secret lives of bacteria: use of *in vivo *expression technology and differential fluorescence induction promoter traps as tools for exploring niche-specific gene expression. Microbiol Mol Biol Rev.

[B19] Moens S, Vanderleyden J (1996). Functions of bacterial flagella. Crit Rev Microbiol.

[B20] Dalton HM, March PE (1998). Molecular genetics of bacterial attachment and biofouling. Curr Opin Biotechnol.

[B21] Lanois A, Jubelin G, Givaudan A (2008). FliZ, a flagellar regulator, is at the crossroads between motility, haemolysin expression and virulence in the insect pathogenic bacterium *Xenorhabdus*. Mol Microbiol.

[B22] Mayeux PR (1997). Pathobiology of lipopolysaccharide. J Toxicol Environ Health.

[B23] De WP, Kwon O, Lin EC (1999). The CpxRA signal transduction system of *Escherichia coli*: growth-related autoactivation and control of unanticipated target operons. J Bacteriol.

[B24] Kato A, Tanabe H, Utsumi R (1999). Molecular characterization of the PhoP-PhoQ two-component system in *Escherichia coli *K-12: identification of extracellular Mg^2+^-responsive promoters. J Bacteriol.

[B25] Soncini FC, Vescovi EG, Groisman EA (1995). Transcriptional autoregulation of the *Salmonella typhimurium *PhoPQ operon. J Bacteriol.

[B26] Groisman EA (2001). The pleiotropic two-component regulatory system PhoP-PhoQ. J Bacteriol.

[B27] Francis MS, Wolf-Watz H, Forsberg A (2002). Regulation of type III secretion systems. Curr Opin Microbiol.

[B28] Wolfe AJ, Parikh N, Lima BP, Zemaitaitis B (2008). Signal integration by the two-component signal transduction response regulator CpxR. J Bacteriol.

[B29] Raivio TL (2005). Envelope stress responses and Gram-negative bacterial pathogenesis. Mol Microbiol.

[B30] Dorel D, Lejeune P, Rodrigue A (2006). The Cpx system of *Escherichia coli*, a strategic signaling pathway for confronting adverse conditions and for settling biofilm communities?. Res Microbiol.

[B31] Brillard J, Ribeiro C, Boemare N, Brehelin M, Givaudan A (2001). Two distinct hemolytic activities in *Xenorhabdus nematophila *are active against immunocompetent insect cells. Appl Environ Microbiol.

[B32] Brillard J, Duchaud E, Boemare N, Kunst F, Givaudan A (2002). The PhlA hemolysin from the entomopathogenic bacterium *Photorhabdus luminescens *belongs to the two-partner secretion family of hemolysins. J Bacteriol.

[B33] Bowen D, Rocheleau TA, Blackburn M, Andreev O, Golubeva E, Bhartia R, ffrench-Constant RH (1998). Insecticidal toxins from the bacterium *Photorhabdus luminescens*. Science.

[B34] ffrench-Constant RH, Bowen D (1999). *Photorhabdus *toxins: novel biological insecticides. Curr Opin Microbiol.

[B35] ffrench-Constant R, Waterfield N, Daborn P, Joyce S, Bennett H, Au C, Dowling A, Boundy S, Reynolds S, Clarke D (2003). *Photorhabdus*: towards a functional genomic analysis of a symbiont and pathogen. FEMS Microbiol Rev.

[B36] Waterfield NR, Bowen DJ, Fetherston JD, Perry RD, ffrench-Constant RH (2001). The tc genes of *Photorhabdus*: a growing family. Trends Microbiol.

[B37] Istivan TS, Coloe PJ (2006). Phospholipase A in Gram-negative bacteria and its role in pathogenesis. Microbiology-Sgm.

[B38] Alfano JR, Collmer A (2004). Type III secretion system effector proteins: Double agents in bacterial disease and plant defense. Ann Rev Phytopathol.

[B39] Buttner D, Bonas U (2003). Common infection strategies of plant and animal pathogenic bacteria. Curr Opin Plant Biol.

[B40] Collmer A, Lindeberg M, Petnicki-Ocwieja T, Schneider DJ, Alfano JR (2002). Genomic mining type III secretion system effectors in *Pseudomonas syringae *yields new picks for all TTSS prospectors. Trends Microbiol.

[B41] Leuzzi R, Serino L, Scarselli M, Savino S, Fontana MR, Monaci E, Taddei A, Fischer G, Rappuoli R, Pizza M (2005). Ng-MIP, a surface-exposed lipoprotein of *Neisseria gonorrhoeae*, has a peptidyl-prolyl cis/trans isomerase (PPIase) activity and is involved in persistence in macrophages. Mol Microbiol.

[B42] Ren P, Rossettini A, Chaturvedi V, Hanes SD (2005). The Ess1 prolyl isomerase is dispensable for growth but required for virulence in *Cryptococcus neoformans*. Microbiology-Sgm.

[B43] Fischer G, Tradler T, Zarnt T (1998). The mode of action of peptidyl prolyl *cis/trans *isomerases in vivo: binding vs. catalysis. FEBS Lett.

[B44] Schiene C, Fischer G (2000). Enzymes that catalyse the restructuring of proteins. Curr Opin Struct Biol.

[B45] Farewell A, Kvint K, Nystrom T (1998). uspB, a new sigma S-regulated gene in *Escherichia coli *which is required for stationary-phase resistance to ethanol. J Bacteriol.

[B46] Bukau B, Horwich AL (1998). The Hsp70 and Hsp60 chaperone machines. Cell.

[B47] Meister A (1988). Glutathione metabolism and its selective modification. J Biol Chem.

[B48] Mannervik B (1987). The enzymes of glutathione metabolism - an overview. Biochem Soc Trans.

[B49] Hayes JD, Flanagan JU, Jowsey IR (2005). Glutathione transferases. Ann Rev Pharmacol Toxicol.

[B50] Morschhauser J, Kohler G, Ziebuhr W, Blum-Oehler G, Dobrindt U, Hacker J (2000). Evolution of microbial pathogens. Philos Trans R Soc Lond B Biol Sci.

[B51] Alamuri P, Maier RJ (2004). Methionine sulphoxide reductase is an important antioxidant enzyme in the gastric pathogen *Helicobacter pylori*. Mol Microbiol.

[B52] Weissbach H, Etienne F, Hoshi T, Heinemann SH, Lowther WT, Matthews B, St John G, Nathan C, Brot N (2002). Peptide methionine sulfoxide reductase: Structure, mechanism of action, and biological function. Arch Biochem Biophys.

[B53] Ikeda T, Laporte DC (1991). Isocitrate dehydrogenase kinase phosphatase - Acek alleles that express kinase but not phosphatase-activity. J Bacteriol.

[B54] Cynkin MA, Ashwell G (1960). Uronic acid metabolism in bacteria. IV. Purification and properties of 2-keto-3-deoxy-D-gluconokinase in *Escherichia coli*. J Biol Chem.

[B55] Delbaere LT, Sudom AM, Prasad L, Leduc Y, Goldie H (2004). Structure/function studies of phosphoryl transfer by phosphoenolpyruvate carboxykinase. Biochim Biophys Acta.

[B56] Camilli A, Mekalanos JJ (1995). Use of recombinase gene fusions to identify *Vibrio cholerae *genes induced during infection. Mol Microbiol.

[B57] Osorio CG, Crawford JA, Michalski J, Martinez-Wilson H, Kaper JB, Camilli A (2005). Second-generation recombination-based in vivo expression technology for large-scale screening for *Vibrio cholerae *genes induced during infection of the mouse small intestine. Infect Immun.

[B58] Sanyal I, Cohen G, Flint DH (1994). Biotin synthase: purification, characterization as a [2Fe-2S] cluster protein, and in vitro activity of the *Escherichia coli *bioB gene product. Biochemistry.

[B59] Travis J, Potempa J, Maeda H (1995). Are bacterial proteinases pathogenic factors. Trends Microbiol.

[B60] Geukens N, De Buck E, Meyen E, Maes L, Vranckx L, Van Mellaert L, Anné J, Lammertyn E (2006). The type II signal peptidase of *Legionella pneumophila*. Res Microbiol.

[B61] Eppinger M, Rosovitz MJ, Fricke WF, Rasko DA, Kokorina G, Fayolle C, Lindler LE, Carniel E, Ravel J (2007). The complete genome sequence of *Yersinia pseudotuberculosis *IP31758, the causative agent of Far East scarlet-like fever. PloS Genet.

[B62] Patzer SI, Hantke K (1998). The ZnuABC high-affinity zinc uptake system and its regulator Zur in *Escherichia coli*. Mol Microbiol.

[B63] Linton KJ, Higgins CF (1998). The *Escherichia coli *ATP-binding cassette (ABC) proteins. Mol Microbiol.

[B64] Heermann R, Fuchs TM (2008). Comparative analysis of the *Photorhabdus luminescens *and the *Yersinia enterocolitica *genomes: uncovering candidate genes involved in insect pathogenicity. BMC Genomics.

[B65] Lazzaroni JC, Germon P, Ray MC, Vianney A (1999). The Tol proteins of *Escherichia coli *and their involvement in the uptake of biomolecules and outer membrane stability. FEMS Microbiol Lett.

[B66] Nikaido H (2003). Molecular basis of bacterial outer membrane permeability revisited. Microbiol Mol Biol Rev.

[B67] Tailliez P, Pages S, Ginibre N, Boemare N (2006). New insight into diversity in the genus *Xenorhabdus*, including the description of ten novel species. Int J Syst Evol Microbiol.

[B68] Grewal PS, Grewal SK, Malik VS, Klein MG (2002). Differences in susceptibility of introduced and native white grub species to entomopathogenic nematodes from various geographic localities. Biol Contr.

[B69] Stock SP, Koppenhöfer AM (2003). *Steinernema scarabaei *n. sp. (Rhabditida: Steinernematidae), a natural pathogen of scarab beetle larvae (Coleoptera: Scarabaeidae) from New Jersey, USA. Nematology.

[B70] Sambrook J, Russell DW, Russell D (2000). Plasmids and their usefulness in molecular cloning. Molecular cloning: A laboratory manual.

[B71] Froussard P (1992). A random-PCR method (rPCR) to construct whole cDNA library from low amounts of RNA. Nucl Acids Res.

[B72] Baltes N, Gerlach GF (2004). Identification of genes transcribed by *Actinobacillus pleuropneumoniae *in necrotic porcine lung tissue by using selective capture of transcribed sequences. Infect Immun.

[B73] Daigle F, Graham JE, Curtiss R (2001). Identification of *Salmonella typhi *genes expressed within macrophages by selective capture of transcribed sequences (SCOTS). Mol Microbiol.

[B74] Graham JE, Clark-Curtiss JE (1999). Identification of *Mycobacterium tuberculosis *RNAs synthesized in response to phagocytosis by human macrophages by selective capture of transcribed sequences (SCOTS). Proc Natl Acad Sci USA.

[B75] Haydel SE, Benjamin WH, Dunlap NE, Clark-Curtiss JE (2002). Expression, autoregulation, and DNA binding properties of the *Mycobacterium tuberculosis *TrcR response regulator. J Bacteriol.

[B76] Hou JY, Graham JE, Clark-Curtiss JE (2002). *Mycobacterium avium *genes expressed during growth in human macrophages detected by selective capture of transcribed sequences (SCOTS). Infect Immun.

[B77] Heck KL, Belle Gv, Simberloff D (1975). Explicit calculation of the rarefaction diversity measurement and the determination of sufficient sample size. Ecology.

[B78] Wang YL, Morse D (2006). Rampant polyuridylylation of plastid gene transcripts in the dinoflagellate Lingulodinium. Nucleic Acids Res.

[B79] Suga K, Mark Welch D, Tanaka Y, Sakakura Y, Hagiwara A (2007). Analysis of expressed sequence tags of the cyclically parthenogenetic rotifer *Brachionus plicatilis*. PLoS ONE.

[B80] Zhu XC, Tu ZJ, Coussens PM, Kapur V, Janagama H, Naser S, Sreevatsan S (2008). Transcriptional analysis of diverse strains *Mycobacterium avium *subspecies paratuberculosis in primary bovine monocyte derived macrophages. Microb Infect.

[B81] Frias-Lopez J, Shi Y, Tyson GW, Coleman ML, Schuster SC, Chisholm SW, DeLong EF (2008). Microbial community gene expression in ocean surface waters. PNAS.

[B82] Baltes N, Buettner FF, Gerlach GF (2007). Selective capture of transcribed sequences (SCOTS) of *Actinobacillus pleuropneumoniae *in the chronic stage of disease reveals an HlyX-regulated autotransporter protein. Vet Microbiol.

[B83] Mazur BJ, Rice D, Haselkorn R (1980). Identification of blue-green-algal nitrogen-fixation genes by using heterologous DNA hybridization probes. Proceedings of the National Academy of Sciences of the United States of America-Biological Sciences.

[B84] Renn SCP, Aubin-Horth N, Hofmann HA (2004). Biologically meaningful expression profiling across species using heterologous hybridization to a cDNA microarray. BMC Genomics.

[B85] Bar-Or C, Bar-Eyal M, Gal TZ, Kapulnik Y, Czosnek H, Koltai H (2006). Derivation of species-specific hybridization-like knowledge out of cross-species hybridization results. BMC Genomics.

[B86] Buckley BA (2007). Comparative environmental genomics in non-model species: using heterologous hybridization to DNA-based microarrays. J Exp Biol.

[B87] Daborn PJ, Waterfield N, Blight MA, ffrench-Constant RH (2001). Measuring virulence factor expression by the pathogenic bacterium *Photorhabdus luminescens *in culture and during insect infection. J Bacteriol.

[B88] Livak KJ, Schmittgen TD (2001). Analysis of relative gene expression data using real-time quantitative PCR and the 2(T)(-Delta Delta C) method. Methods.

