# Supplementary figures and images for: Comparative in vivo gene expression of the closely related bacteria Photorhabdus temperata and Xenorhabdus koppenhoeferi upon infection of the same insect host, Rhizotrogus majalis
Source: BMC Genomics. 2009 Sep 15;10:433. doi: 10.1186/1471-2164-10-433 (PMC2760582; doi:10.1186/1471-2164-10-433)

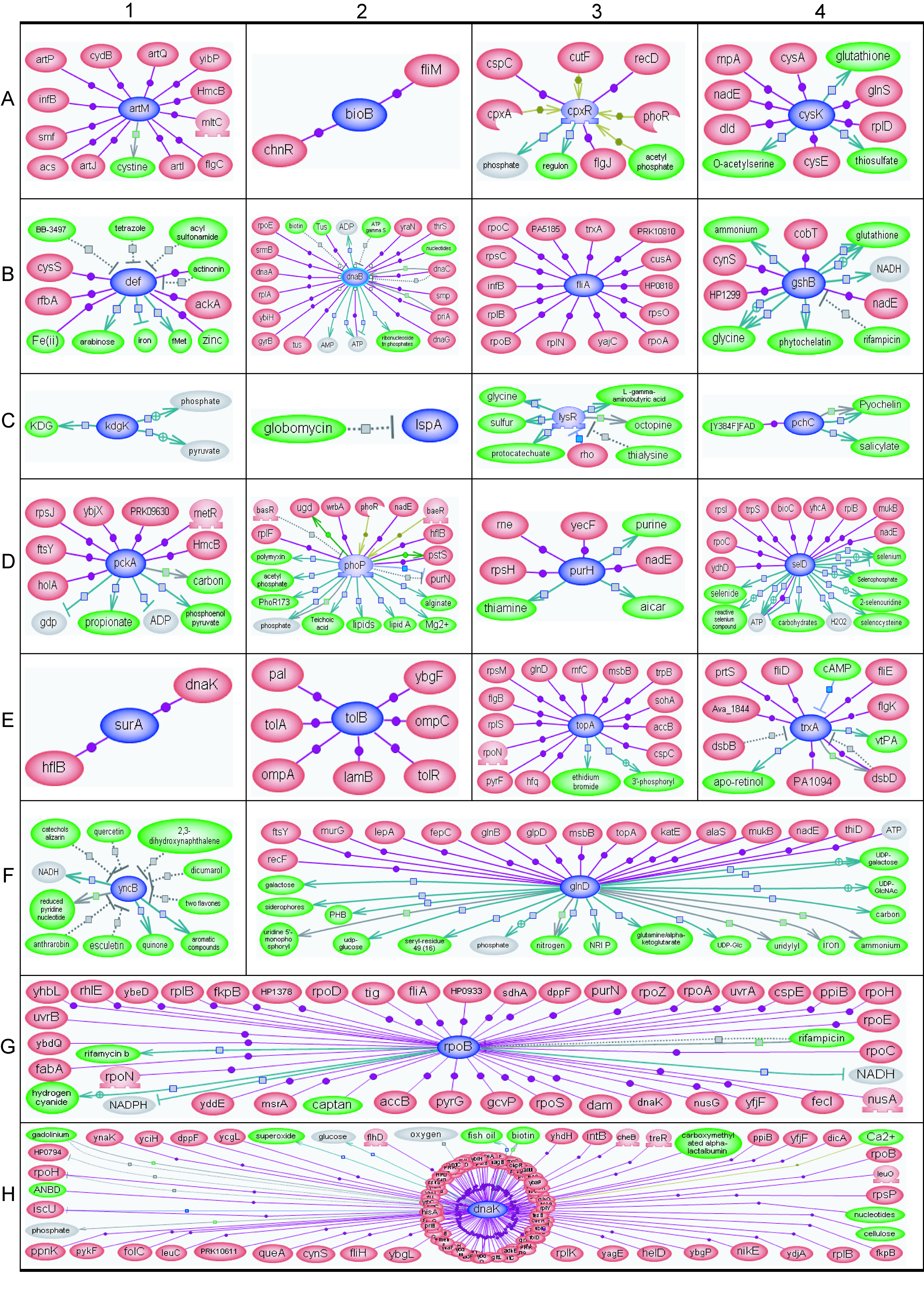

Supplement: Additional file 1 — Direct linkages of proteins and small molecules to the SCOTS identified Photorhabdus temperata genes induced upon infection of Rhizotrogus majalis. Linkages of molecules to identified genes were built in the PathwayStudio program. Gray and green ovals indicate small molecules. The gene products are represented by red or blue ovals, where blue ovals indicate genes identified in this study and red ovals represent genes in the database of the PathwayStudio program. The relationships are indicated by lines as follows: Binding - violet links with violet circles, MolTransport - gray arrows with green rectangles, MolSynthesis - blue arrows with blue rectangles, ProtModification - brown arrows, Regulation - gray links with gray rectangles, PromoterBinding - green arrows with green circles, and Expression - blue arrows with blue rectangles. Arrows with "+" indicate positive regulation and with "-" indicate negative regulation. [file 1471-2164-10-433-S1.tiff]

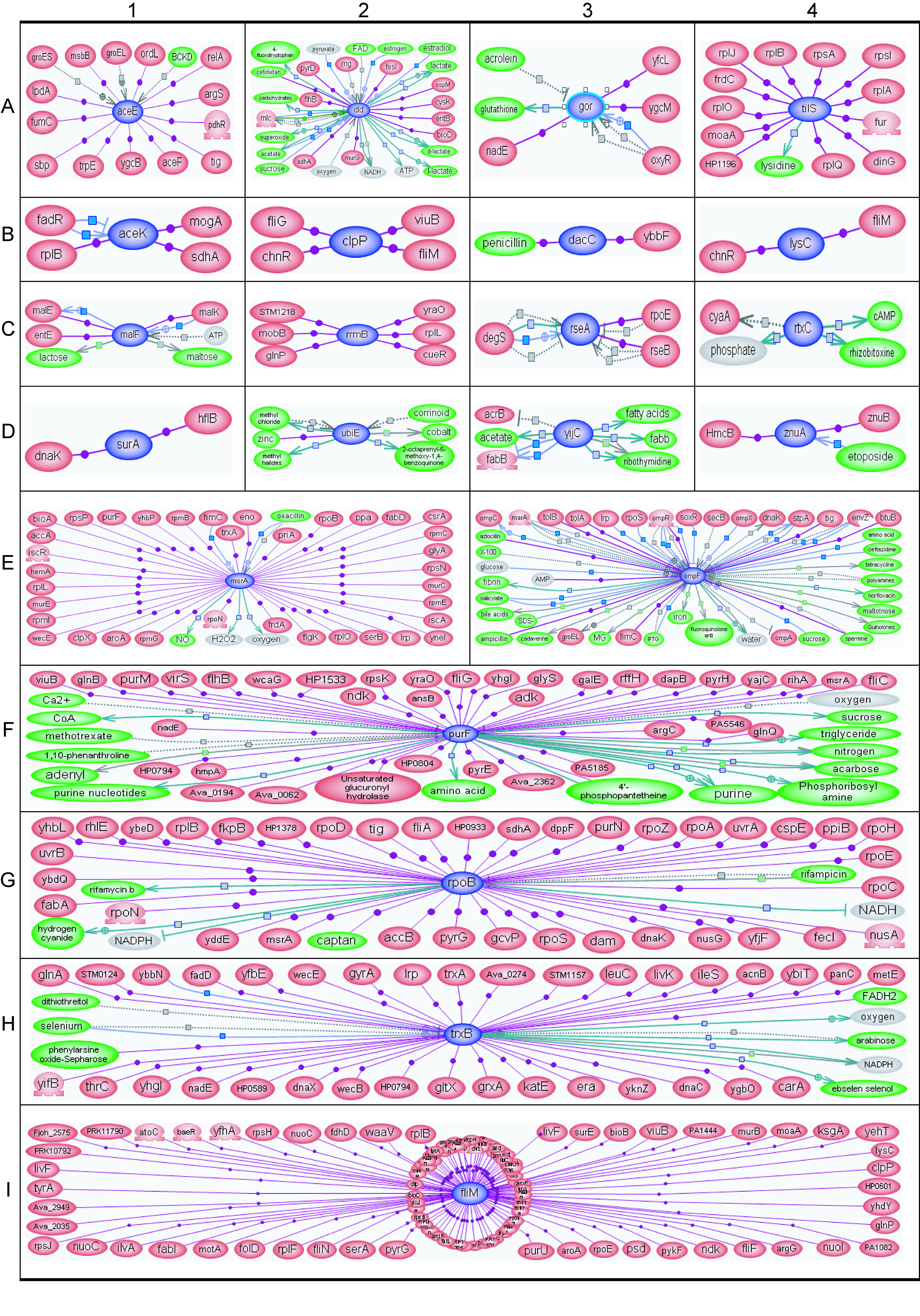

Supplement: Additional file 2 — Direct linkages of proteins and small molecules to the SCOTS identified Xenorhabdus koppenhoeferi genes induced upon infection of Rhizotrogus majalis. Linkages of molecules to identified genes were built in the PathwayStudio program. Gray and green ovals indicate small molecules. The gene products are represented by red or blue ovals, where blue ovals indicate genes identified in this study and red ovals represent genes in the database of the PathwayStudio program. The relationships are indicated by lines as follows: Binding - violet links with violet circles, MolTransport - gray arrows with green rectangles, MolSynthesis - blue arrows with blue rectangles, ProtModification - brown arrows, Regulation - gray links with gray rectangles, PromoterBinding - green arrows with green circles, and Expression - blue arrows with blue rectangles. Arrows with "+" indicate positive regulation and with "-" indicate negative regulation. [file 1471-2164-10-433-S2.tiff]

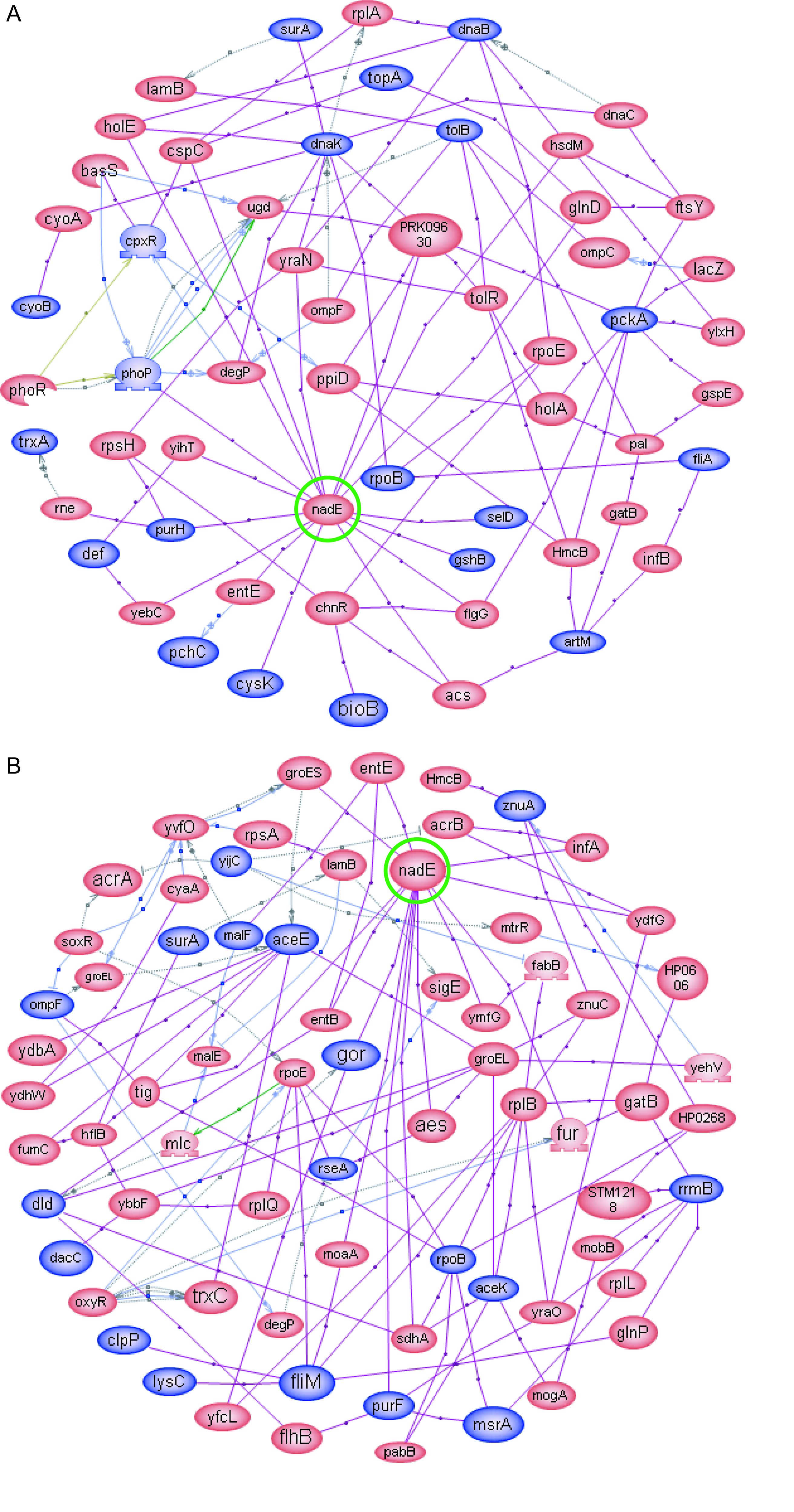

Supplement: Additional file 3 — The network interactions between gene products of Photorhabdus temperata (panel A) and Xenorhabdus koppenhoeferi (panel B) built by leveraging databases of published literature. The gene expression data obtained in this study were analyzed and visualized by PathwayStudio program. The gene products are represented by ovals, where blue ovals represent products encoded by genes induced in P. temperata (panel A) and X. koppenhoeferi (panel B) upon infection of the insect Rhizotrogus majalis and red ovals represent genes in the database of the PathwayStudio program. The product encoded by gene nadE was highlighted with a green circle as this gene was common to both bacteria and interacts with many other gene products in both networks. The relationships between gene products are indicated by lines as follows: Binding - violet links with violet circles, MolTransport - gray arrows with green rectangles, MolSynthesis - blue arrows with blue rectangles, ProtModification - brown arrows, Regulation - gray links with gray rectangles, PromoterBinding - green arrows with green circles, and Expression - blue arrows with blue rectangles. Arrows with "+" indicate positive regulation and with "-" indicate negative regulation. [file 1471-2164-10-433-S3.tiff]
