# Supplementary material for: Comparative in vivo gene expression of the closely related bacteria Photorhabdus temperata and Xenorhabdus koppenhoeferi upon infection of the same insect host, Rhizotrogus majalis
Source: BMC Genomics. 2009 Sep 15;10:433. doi: 10.1186/1471-2164-10-433 (PMC2760582; doi:10.1186/1471-2164-10-433)
Supplement: Additional file 4 — Supplementary Table 1. Oligonucleotide sequences used for quantitative real-time PCR analyses. [file 1471-2164-10-433-S4.pdf]

Table S1: Oligonucleotide sequences used for quantitative real-time PCR analyses

Gene	Forward primer (5’ - 3’)	Reverse primer (5’ – 3’)
cysK	TAATACCGGGATTGCGCTTGCCTA	ATGGCGCCTTTCATACCTTTAGCG
def	ACACGGCCTTCTTCAATCCCAGTA	ATGTACGTAGAGGAAGGCATTGGC
dnaB	CCTGCAATTAATGCGGGTTCCATC	TACGGTTGAGCTGAGAAAGTGCGA
lysR	AGAAGGGCGGTGAATGGGTTTGAT	GGAAGGCATCGGCGTTTCTATTGT
pchC	AGAAGCTGCGAGAATGCCGATAGT	GCCATCACCTGGCAATCAATCCAT
pckA	ATTGTCCGTGATGATGTCACCCGT	CGCACCACAGAAAGCATCGACAAT
phoP	ATGGTTTGAGTCTGATCCGTCGCT	GCCTGCATCCGGGCAATAATTTCT
ptst1	GATTTCGCCGATGGCGATACTACT	TGGCAGTTTGCCTTGTAGGA
sctL	CAAACCCAGCTTCAGTGTCAGCAA	TGTTGCCGCCTGTTCAGGATTAAC
selD	AAATGACAGCGCTTCAGCGTAACC	TCAGAACTCTGCCTGACTGTTGCT
tolB	AGCTGGCGTTTGCCCTGTCTAAAT	AGACCAAAGTCTGGTTATCCGGCA
virB	TTATTTCGGGTGGTGCCGTCTAGT	AAGAGATCCTCATCACGCCCATCT
**16SPt**	AGCGCAACCCTTATCCTTTGTTGC	TCGCGAGGTCGCTTCACTTTGTAT
aceK	TGGTCTGAGCCAAGATCACAGTTG	ACTGATGAGGAAATCTGGGCGCTT
clpP	TCAGCCATTAGGTGGTTTCCAAGG	CACGTTCAGTGTCCTGTGCAATCT
cobJ	AAAGCGCAATGTGGAAGTCAGGCT	CGCGGATTGTAGAAACAGACCACA
dacC	TCCGAAGTAGCTTTGGGAGTGGAA	GCCTCTTTAGCAACCAATGGACAC
dld	CGTCGCCACATTCTGTCTACTTTCCA	AAATGGCATCCATCCGCCCTTTCA
malF	GGGATAGCCCAACCATGTATTTACG	CTGGTGCTGAATGGGTTGTTTGGT
res	AATTTCAGTGCGACTCCTTTGCGG	TCAATTTCCTTCCCGTCTTCCCGA
rtxC	TGGTATTTCCGTCAATGTGGCGTG	GGGCATAAGAGGCGTTGATGTTCA
tilS	TTATGAGCCACTTCTGACGGCACT	CGTCACGTAAGCGAACCAGCAAAT
virH	ACTGACCCGATTATTACAGCGCCT	TGCAATACCACGCTCATGTTTCGC
xhlA	GTACCCTGCAAAGCTTCAGCACTT	TGACGTAACGACCTGACCATTGCT
xkst4	AGCTGGTAGAGCATCTCCCTTACA	ACCAACTGAGCTAATCGCCCAATC
**16SXk**	AGCGCAACCCTTATCCTTTGTTGC	TCGCGAGGTCGCTTCTCTTTGTAT
